# H2A ubiquitination is essential for Polycomb Repressive Complex 1-mediated gene regulation in *Marchantia polymorpha*

**DOI:** 10.1186/s13059-021-02476-y

**Published:** 2021-09-01

**Authors:** Shujing Liu, Minerva S. Trejo-Arellano, Yichun Qiu, D. Magnus Eklund, Claudia Köhler, Lars Hennig

**Affiliations:** 1 Department of Plant Biology, Swedish University of Agricultural Sciences and Linnean Center for Plant Biology, 75007 Uppsala, Sweden; 2 Present address: Department of Cell and Developmental Biology, John Innes Centre, Norwich, NR4 7UH UK; 3 Max Planck Institute of Molecular Plant Physiology, Potsdam, 14476 Potsdam-Golm, Germany; 4 Department of Plant Ecology and Evolution, Evolutionary Biology Centre, Uppsala University, 75236 Uppsala, Sweden

**Keywords:** H2Aub, H3K27me3, Gene regulation, Polycomb, PRC1, *Marchantia polymorpha*

## Abstract

**Background:**

Polycomb repressive complex 1 (PRC1) and PRC2 are chromatin regulators maintaining transcriptional repression. The deposition of H3 lysine 27 tri-methylation (H3K27me3) by PRC2 is known to be required for transcriptional repression, whereas the contribution of H2A ubiquitination (H2Aub) in the Polycomb repressive system remains unclear in plants.

**Results:**

We directly test the requirement of H2Aub for gene regulation in *Marchantia polymorpha* by generating point mutations in H2A that prevent ubiquitination by PRC1. These mutants show reduced H3K27me3 levels on the same target sites as mutants defective in PRC1 subunits MpBMI1 and the homolog MpBMI1L, revealing that PRC1-catalyzed H2Aub is essential for Polycomb system function. Furthermore, by comparing transcriptome data between mutants in MpH2A and MpBMI1/1L, we demonstrate that H2Aub contributes to the PRC1-mediated transcriptional level of genes and transposable elements.

**Conclusion:**

Together, our data demonstrates that H2Aub plays a direct role in H3K27me3 deposition and is required for PRC1-mediated transcriptional changes in both genes and transposable elements in *Marchantia*.

**Supplementary Information:**

The online version contains supplementary material available at 10.1186/s13059-021-02476-y.

## Background

Polycomb group (PcG) proteins are evolutionarily conserved epigenetic regulators which maintain transcriptional gene repression in essential cellular and developmental processes in eukaryotes [1–4]. PcG proteins typically belong to one of the two functionally distinct multi-protein complexes: Polycomb Repressive Complex 1 (PRC1) and PRC2. PRC1 promotes chromatin compaction and catalyzes mono-ubiquitination on histone 2A (H2Aub) mainly at lysine 119 in mammals, lysine 118 in *Drosophila*, and lysine 121 in *Arabidopsis* [4–8], whereas PRC2 tri-methylates histone 3 at lysine 27 (H3K27me3) [9–12]. The catalytic core of the mammalian PRC1 is composed of the E3 ubiquitin ligases RING1A or RING1B and one of six Polycomb RING finger (PCGF) proteins [13–15], while in *Drosophila* it consists of RING1 (encoded by the *Sce* gene) and one of two PCGF proteins: Psc or Su(z)2 [16–18]. The *Arabidopsis* PRC1 core includes AtRING1A or AtRING1B and one of the three AtBMI1s (homologs of PCGF4) [6, 19–21].

Previous studies on the Polycomb repressive system in *Drosophila* and mammals first proposed a PRC2-initiated hierarchical model where PRC2 establishes H3K27me3, which is then recognized by chromodomain-containing subunits of the canonical PRC1 (cPRC1). Nevertheless, later studies found this classical hierarchical model not sufficient to explain the Polycomb repressive system [13, 22, 23]. Instead, it was found that non-canonical PRC1 (ncPRC1) lacking chromodomain-containing subunits can recruit PRC2 and establish stable Polycomb repressive domains [24–27]. This data points that the PRC1 catalytic function is required for PRC2 recruitment, which was supported by recent work showing that in mouse embryonic stem cells (ESCs), loss of RING1B catalytic activity largely phenocopies the complete removal of the RING1B protein [28, 29]. Nevertheless, whether this is a generally applicable concept remains to be established. In *Drosophila*, H2AK118ub seems not required for repression of Polycomb target genes during the early stages of embryo development and PRC2 binding to chromatin requires PRC1 but not H2Aub [30, 31]. Similarly, during neuronal fate restriction in mouse, PRC1 repression was shown to function independently of ubiquitination [32]. This data suggests that there are developmental context-specific differences in the functional requirement of the catalytic activity of PRC1.

PRC1-catalyzed H2Aub has been intensively studied in *Arabidopsis thaliana*. H2Aub level, H3K27me3 incorporation and chromatin accessibility were shown to be affected by the depletion of components of PRC1 in *Arabidopsis* [33–35]. Nevertheless, it remains unclear thus far whether H2Aub is required for H3K27me3 targeting. PRC1 is composed of multiple proteins that engage in interactions with PRC2 components. Thus, AtRING and AtBMI1 in PRC1 can interact with LHP1, which co-purifies with PRC2 in *Arabidopsis* [19, 20, 36], suggesting that PRC1 rather than H2Aub promotes H3K27me3 by interacting and recruiting PRC2 to chromatin. H2Aub is associated with permissively accessible chromatin and the average transcription levels of only-H2Aub marked genes are higher than that of H2Aub/H3K27me3 and only-H3K27me3 marked genes in *Arabidopsis* [33, 35]. Consistently, removal of H2Aub is required for stable repression of Polycomb target genes [34]. Considering these studies proposing an activating role of H2Aub together with the fact that H2Aub is not required for Polycomb-mediated repression in *Drosophila* and neuronal fate restriction of mouse, it remains to be investigated whether H2Aub is indeed essential for PRC1-mediated gene repression. Thus, to directly test the functional role of H2Aub, we generated H2Aub mutants by replacing the endogenous H2A by H2A variants with mutated lysines in the liverwort *Marchantia polymorpha*.

*Marchantia* shares many signaling pathways with *Arabidopsis* and other seed plants [37]. Together with its low genetic redundancy and possibilities to easily generate mutants, *Marchantia* is an ideal plant model to study the evolutionarily conserved Polycomb system. There is only a single gene encoding canonical H2A in *Marchantia*, compared to four genes in *Arabidopsis* [38]. We generated lysine to arginine substitutions in H2A on residues K115/116/119 and demonstrate that all three lysines are ubiquitinated in vivo and likely have redundant functions. We furthermore show that H2Aub mediates H3K27me3 incorporation in both genes and transposable elements (TEs) in *Marchantia* and reveal that H2Aub is essential for both PRC1-mediated transcriptional activation and silencing.

## Results

### H2Aub mediates H3K27me3 deposition on Polycomb target sites in genes and TEs

**Fig. 1:**
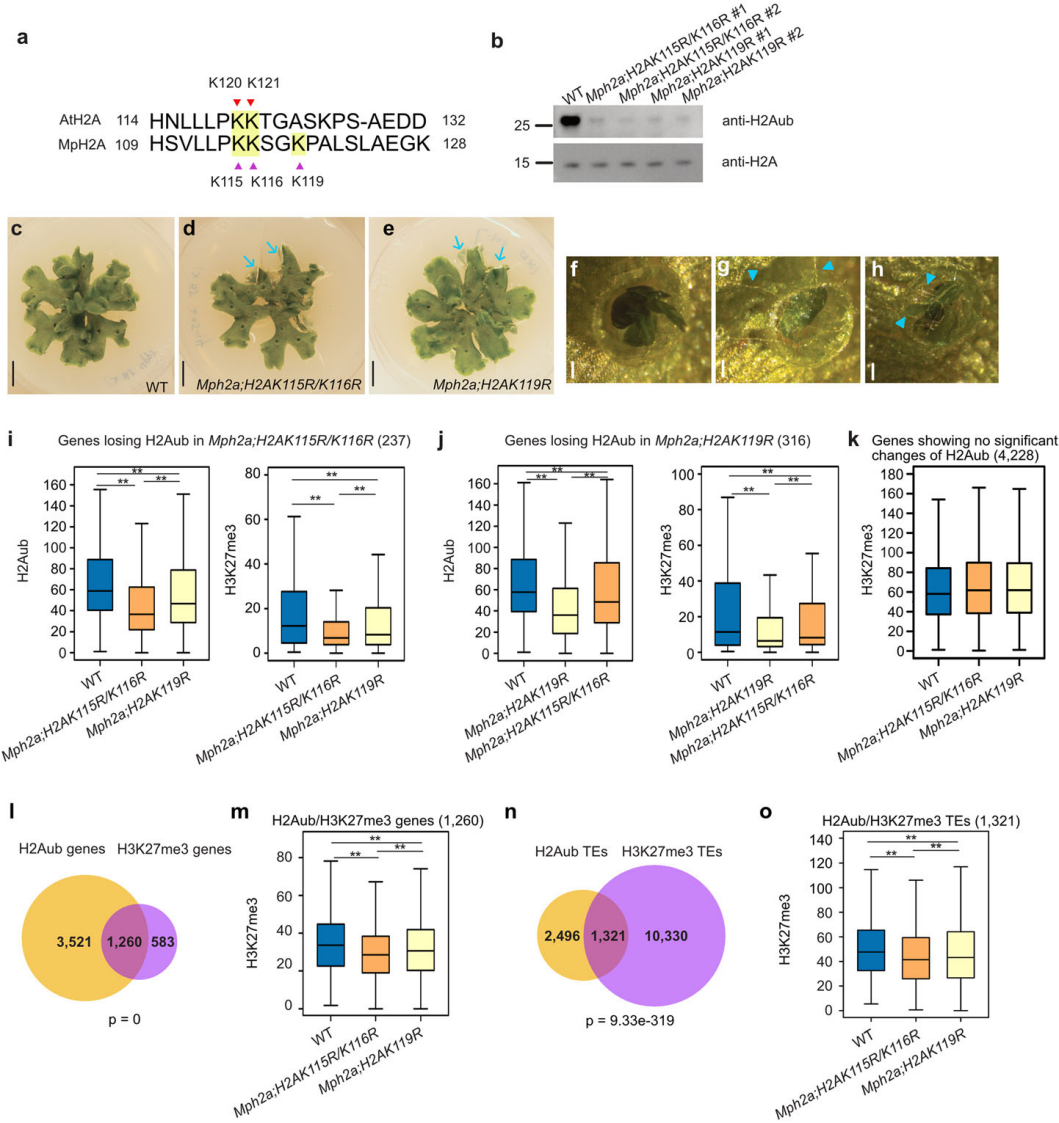
H2Aub directly contributes to the deposition of H3K27me3. **a** Lysine residues in *Arabidopsis* and *Marchantia* H2A. Lysine 120 (K120) and K121 of H2A in *Arabidopsis* and potential ubiquitination sites (K115, K116, and K119) in H2A of *Marchantia* are highlighted. **b** Western blot of bulk H2Aub and H2A in wild type (WT), *Mph2a;H2AK115R/K116R* #1, *Mph2a;H2AK115R/K116R* #2, *Mph2a;H2AK119R* #1, and *Mph2a;H2AK119R* #2 mutants. **c–e** Phenotypes of 28-day-old WT, *Mph2a;H2AK115R/K116R* #1, and *Mph2a;H2AK119R* #1 mutants. Blue arrows point to downward curled edges of the thallus. Scale bars, 1 cm. **f–h** Gemma cup phenotypes of WT, *Mph2a;H2AK115R/K116R* #1, and *Mph2a;H2AK119R* #1 mutants. Blue arrowheads point at visible rhizoids produced by non-dormant gemmae in the mutants. Scale bars, 0.05 cm. **i** Boxplots showing H2Aub and H3K27me3 levels (RPKM, reads per kilobase per million mapped reads) of genes losing H2Aub in *Mph2a;H2AK115R/K116R* in WT, *Mph2a;H2AK115R/K116R*, and *Mph2a;H2AK119R* mutants. **j** Boxplots showing H2Aub and H3K27me3 levels (RPKM) of genes losing H2Aub in *Mph2a;H2AK119R* in WT, *Mph2a;H2AK119R*, and *Mph2a;H2AK115R/K116R* mutants. **k** Boxplot showing H3K27me3 levels (RPKM) of genes that do not show significant changes of H2Aub in *h2a_ub* mutants in WT, *Mph2a;H2AK115R/K116R*, and *Mph2a;H2AK119R* mutants. H2Aub and H3K27me3 levels in i-k were calculated as the average RPKM from 1 kb upstream of the transcriptional start to the transcriptional end of genes. **l** Venn diagram showing overlap of H2Aub-marked genes and H3K27me3-marked genes in wild type (WT). Significance was tested using a hypergeometric test. **m** Boxplot showing H3K27me3 levels of H2Aub/H3K27me3 genes in WT, *Mph2a;H2AK115R/K116R*, and *Mph2a;H2AK119R* mutants. H3K27me3 levels were calculated as the average RPKM from 1 kb upstream of the transcriptional start to the transcriptional end of genes. **n** Venn diagram showing overlap of H2Aub-marked transposable elements (TEs) and H3K27me3-marked TEs in WT. **o** Boxplot showing H3K27me3 levels of H2Aub/H3K27me3 TEs in WT, *Mph2a;H2AK115R/K116R*, and *Mph2a;H2AK119R* mutants. H3K27me3 levels were calculated as the average RPKM from the start to the end of TEs. Boxes show medians and the interquartile range, and error bars show the full range excluding outliers. **p < 0.01 (Wilcoxon test)

In mutants of PRC1 components, decreased H2Aub correlates with reduced H3K27me3 in *Arabidopsis* [33, 35]. To elucidate the functional requirement of H2Aub to induce H3K27me3 incorporation, we generated H2Aub depleted lines by introducing point mutations in the potential ubiquitination sites of canonical MpH2A (Additional file 1: Figure S1a). We co-transformed the point mutated *H2A* variants and a CRISPR construct designed to knock out the endogenous *H2A* (Additional file 1: Figure S1b, c). Lysine 120 (K120) and K121 of *Arabidopsis* H2A were shown to be ubiquitinated by AtBMI1 in vitro [6, 20], corresponding to K115 and K116 in MpH2A (Fig. 1a). In *Drosophila*, mutations of four close lysine sites (K117, K118, K121 and K122) are required to abolish total H2Aub [30]. We therefore generated *Mph2a;H2AK115R/K116R* and *Mph2a;H2AK119R* mutants (jointly referred to as *h2a_ub* mutants) by substituting the C-terminal lysine residues K115 and K116 or K119 of MpH2A with arginine. We failed to obtain *Mph2a* mutants expressing *H2A* variants with all three point mutations, indicating that the three lysine sites of MpH2A are functionally redundant. The global H2Aub level was strongly decreased in lines of *Mph2a;H2AK115R/K116R* and *Mph2a;H2AK119R* mutants compared to wild type (WT) (Fig. 1b). Nevertheless, there was a remaining H2Aub signal in both mutant lines, indicating that all lysine residues can be ubiquitinated in vivo and likely act redundantly. The most obvious defects of *h2a_ub* mutants were downward curled edges of the thallus that grew into the growth media and decreased gemmae dormancy compared to WT (Fig. 1c–h).

To understand the connection between H2Aub and H3K27me3 in the Polycomb repressive system, we generated ChIP-seq data for H3, H2Aub, and H3K27me3 in WT, *Mph2a;H2AK115R/K116R* #1, and *Mph2a;H2AK119R* #1 mutants. To validate our ChIP-seq data, we compared the H3K27me3 peaks in our WT with previously published data [39]. The majority of peaks overlapped between both datasets (Additional file 1: Figure S2), supporting the quality of our data. We found that genes with decreased H2Aub in the *Mph2a;H2AK115R/K116R* mutant had also decreased H2Aub and H3K27me3 levels in the *Mph2a;H2AK119R* mutant, but to a lesser extent (Fig. 1i, Additional file 1: Figure S3a, c). Decreased levels of H2Aub and H3K27me3 occurred over the whole length of genes, including the 1 kb upstream and downstream regions (Additional file 1: Figure S3a). Conversely, genes with reduced H2Aub level in the *Mph2a;H2AK119R* mutant were less affected in the *Mph2a;H2AK115R/K116R* mutant (Fig. 1j, Additional file 1: Figure S3b, d), supporting the notion that all three lysine residues of MpH2A are targeted by ubiquitination and H3K27me3 deposition is affected upon H2Aub depletion. In those H2Aub marked genes that did not undergo changes of H2Aub in either *h2a_ub* mutant, also the level of H3K27me3 level did not obviously change (Fig. 1k). We further analyzed H3K27me3 on genes marked by both H2Aub and H3K27me3 (H2Aub/H3K27me3, overlapped genes in Fig. 1l) and found H3K27me3 levels to be decreased in both *h2a_ub* mutants compared to WT (Fig.1m, Additional file 1: Figure S3e). Due to compensated ubiquitination of K115/K116 and K119 in H2A, decreased H2Aub was only observed in the promoter region of H2Aub/H3K27me3 gene (Additional file 1: Figure S3f). Reduced H3K27me3 in the gene body of H2Aub/H3K27me3 genes implies that the ubiquitination on K115/K116 and K119 in H2A is not completely functionally redundant. H3K27me3 was also decreased on only-H3K27me3 marked genes (*n*=583), correlating with mildly increased levels of H2Aub on the 1 kb promoter and highly increased levels of H2Aub on the gene body of only-H3K27me3 genes in *h2a_ub* mutants (Additional file 1: Figure S3g, h). Gain of H2Aub is associated with the recruitment of REF6 that mediates H3K27me3 demethylation in *Arabidopsis* [34], providing an explanation for the loss of H3K27me3 on only-H3K27me3 genes.

In *Marchantia*, 60% of H3K27me3 peaks are present in intergenic regions [39]; however, the location of H2Aub peaks remains to be explored. Out of 6575 H2Aub peaks identified in WT, about 20% mapped to intergenic regions, while most of the H2Aub peaks were located in gene body or promoter regions (Additional file 1: Figure S4). We found H2Aub and H3K27me3 peaks to be enriched on TEs (Fig. 1n), especially the H3K27me3-only peaks. TEs covered by H2Aub and H3K27me3 had also decreased H3K27me3 levels in both *h2a_ub* mutants (Fig. 1o), revealing that H2Aub is required to recruit H3K27me3 to TE regions in plants. To understand whether H3K27me3 is affected genome-wide in *h2a_ub* mutants, we tested H3K27me3 levels on all genes and all TEs. H3K27me3 was globally decreased on genes but not on TEs (Additional file 1: Figure S5), consistent with H2Aub mainly targeting genic region.

### H2Aub contributes to transcriptional repression and activation

**Fig. 2:**
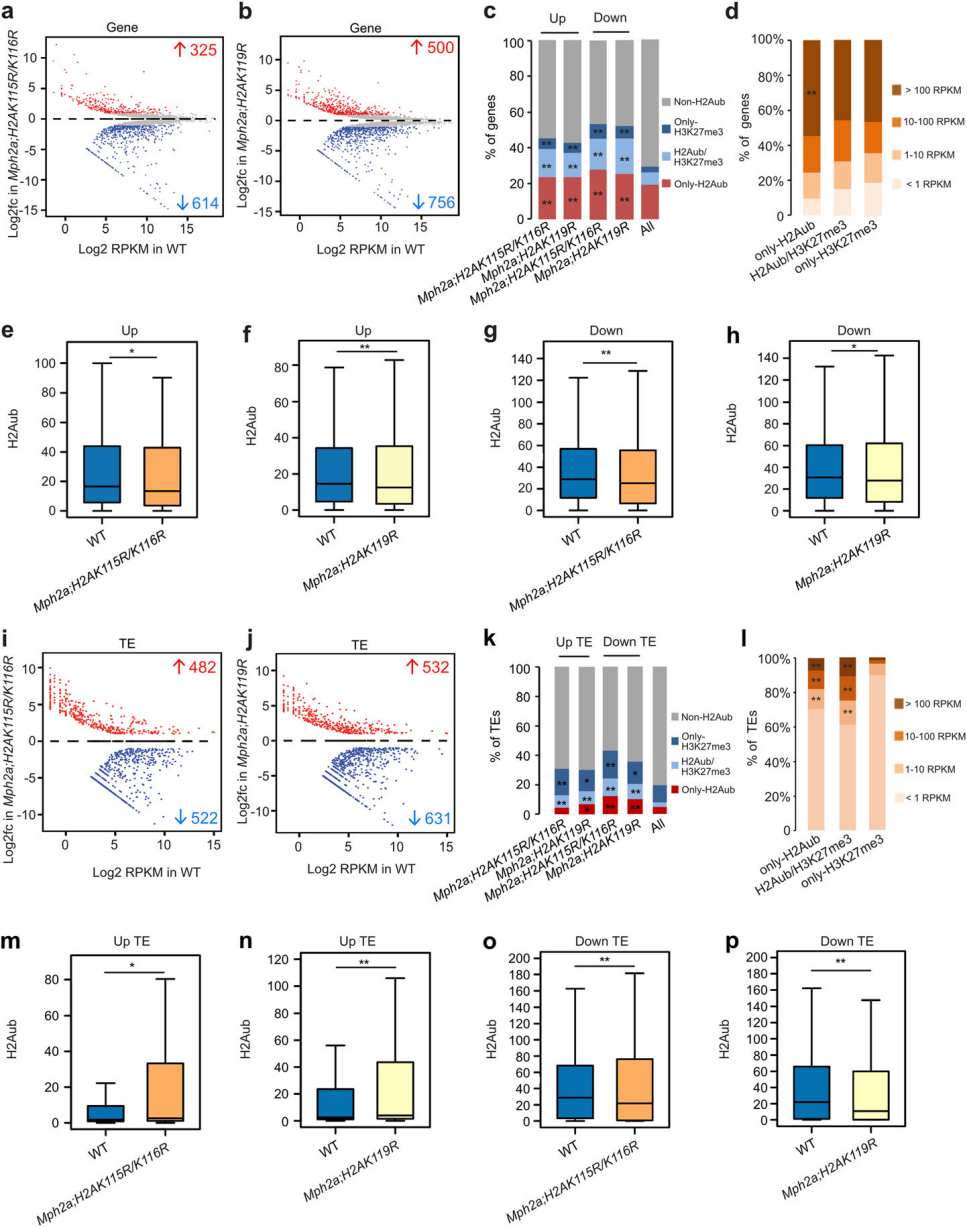
H2Aub is required for gene repression and activation. **a**, **b** MA plots showing differential gene expression (Log2 fold change (Log2fc)) in *Mph2a;H2AK115R/K116R* (**a**) and *Mph2a;H2AK119R* (**b**) mutants compared to wild type (WT). Significant changes are marked in red (log2 fold change ≥ 1 and adjusted *p* value ≤ 0.05) and blue (log2 fold change ≤ −1 and adjusted *p* value ≤ 0.05). **c** Presence of only-H2Aub, H2Aub/H3K27me3, or only-H3K27me3 marks on upregulated (Up) and downregulated (Down) genes in *Mph2a;H2AK115R/K116R* and *Mph2a;H2AK119R* mutants. Presence of modifications is based on their distribution in WT. **p < 0.01 (Hypergeometric test). **d** Expression level distribution of genes marked by only-H2Aub, H2Aub/H3K27me3, and only-H3K27me3 in WT. **p < 0.01 (Hypergeometric test). **e, f** Boxplots showing the H2Aub level (RPKM, reads per kilobase per million mapped reads) on upregulated genes in *Mph2a;H2AK115R/K116R* (e) and *Mph2a;H2AK119R* (f) mutants. **g, h** Boxplots showing H2Aub levels on downregulated genes in *Mph2a;H2AK115R/K116R* (**g**) and *Mph2a;H2AK119R* (**h**) mutants. H2Aub levels in e-h were calculated as the average RPKM from 1 kb upstream of the transcriptional start to the transcriptional start of genes. *p < 0.05; **p < 0.01 (Wilcoxon test). **i, j** MA plots showing differential expression of transposable elements (TEs) (Log2fc) in *Mph2a;H2AK115R/K116R* (**i**) and *Mph2a;H2AK119R* (**j**) mutants. Significant gene expression changes are marked in red (log2 fold change ≥ 1 and adjusted *p* value ≤ 0.05) and blue (log2 fold change ≤ −1 and adjusted *p* value ≤ 0.05). **k** Presence of only-H2Aub, H2Aub/H3K27me3, or only-H3K27me3 marks on upregulated (Up TE) and downregulated (Down TE) TEs in *Mph2a;H2AK115R/K116R* and *Mph2a;H2AK119R* mutants. *p < 0.05; **p < 0.01 (Hypergeometric test). **l** Expression level distribution of transposable elements (TEs) marked by only-H2Aub, H2Aub/H3K27me3, and only-H3K27me3 in WT. **p < 0.01 (Hypergeometric test). **m**, **n** Boxplots showing the H2Aub levels on upregulated TEs in *Mph2a;H2AK115R/K116R* (**m**) and *Mph2a;H2AK119R* (**n**) mutants. **o**, **p** Boxplots showing H2Aub levels on downregulated TEs in *Mph2a;H2AK115R/K116R* (**o**) and *Mph2a;H2AK119R* (**p**) mutants. H2Aub levels in m-p were calculated as the average RPKM from the start to the end of TEs. Boxes show medians and the interquartile range, and error bars show the full range excluding outliers. *p < 0.05; **p < 0.01 (Wilcoxon test)

Although many transcriptionally active genes are marked with H2Aub and H2Aub is associated with a permissive chromatin state in *Arabidopsis* [33–35], it is unknown whether H2Aub is required for gene activation. We noted that in *h2a_ub* mutants there were more downregulated than upregulated genes (Fig. 2a, b), suggesting that H2Aub has an activating role for gene expression in *Marchantia*. Both upregulated and downregulated genes in *h2a_ub* mutants were enriched for genes with H2Aub and H3K27me3 marks (Fig. 2c). The only-H2Aub marked genes had a higher number of highly transcriptional active genes compared to H2Aub/H3K27me3 and only-H3K27me3 genes (Fig. 2d). We tested the H2Aub level on upregulated and downregulated genes in *h2a_ub* mutants and found that H2Aub levels were decreased in the promoter regions but not in the gene body region of both gene categories (Fig. 2e–h and Additional file 1: Figure S6a-d). Since the accessibility of promoter regions is essential for transcription factor binding, we propose that decreased H2Aub in promoter regions contributes to gene repression as well as activation. We also found more downregulated than upregulated TEs in *h2a_ub* mutants (Fig. 2i, j), indicating a role of H2Aub in TE activation. H2Aub and H3K27me3 marked TEs were enriched among deregulated TEs (Fig. 2k). The only-H2Aub and H2Aub/H3K27me3 marked TEs were more frequently highly transcriptionally active than the only-H3K27me3 marked TEs (Fig. 2l), pointing that H2Aub has the potential to activate TE expression and that removal of H2Aub is required for stable repression. We found significantly decreased H2Aub levels only on downregulated TEs, while upregulated TEs had very low levels of H2Aub (Fig. 2m–p, Additional file 1: Figure S6i, j), supporting the notion that H2Aub has an activating role towards TE transcription. Downregulated TEs had also decreased levels of H3K27me3 (Additional file 1: Figure S6k, l), suggesting that loss of H3K27me3 is not sufficient for TE activation.

### MpBMI1/1L regulate morphological development of *Marchantia* and are involved in gene repression and activation

**Fig. 3:**
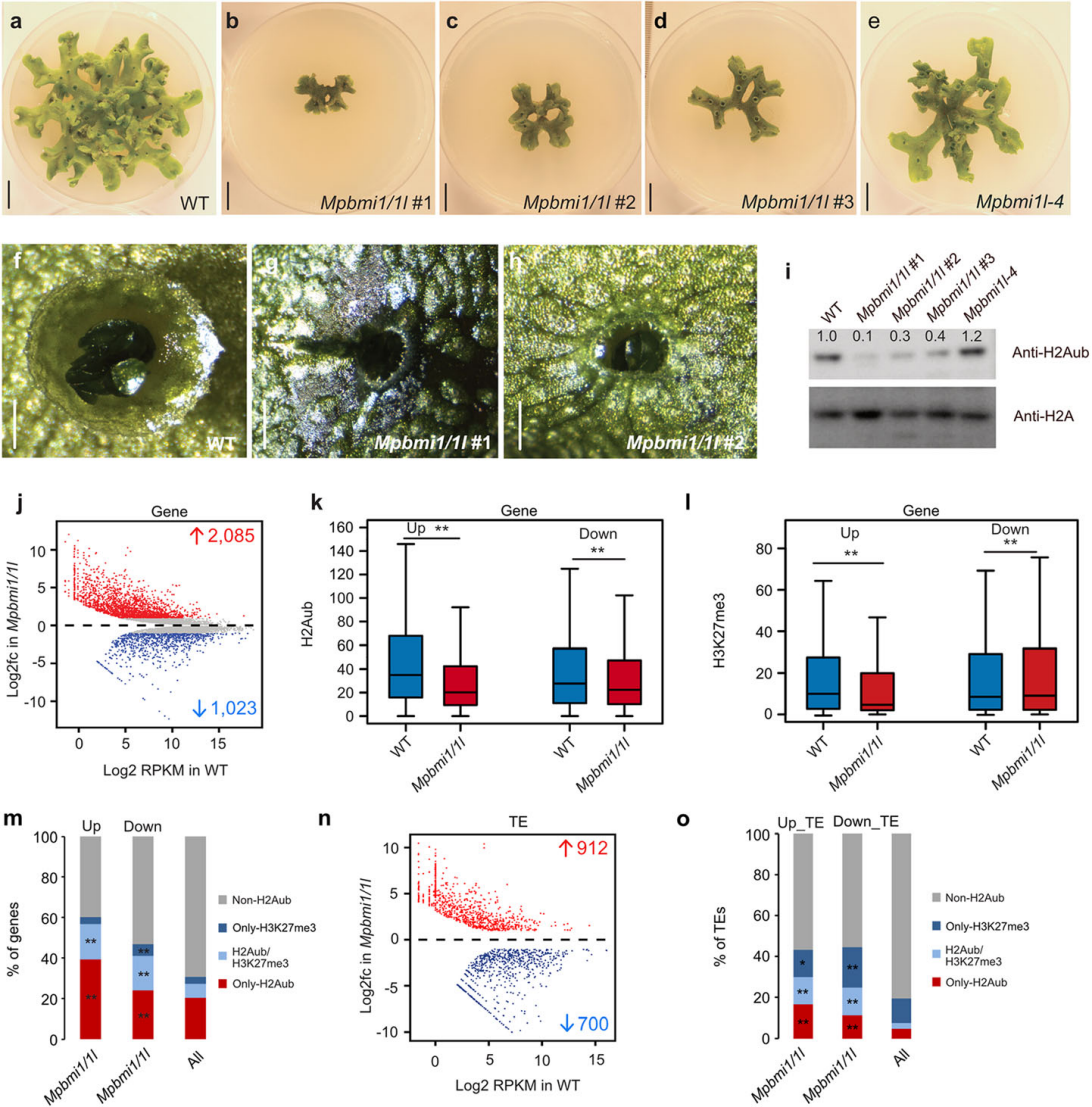
MpBMI1/1L affect morphological development of *Marchantia* and regulate gene silencing and activation. **a–e** Morphological phenotypes of 35-day-old wild type (WT), *Mpbmi1/1l* #1, *Mpbmi1/1l* #2, *Mpbmi1/1l* #3, and *Mpbmi1l-4* lines. Scale bars: 1 cm. **f–h** Gemma cups of 35-day-old WT, *Mpbmi1/1l* #1, and *Mpbmi1/1l* #2 lines. Scale bars: 0.1 cm. **i** Western blot showing the bulk H2Aub levels in WT, *Mpbmi1/1l* #1, *Mpbmi1/1l* #2, *Mpbmi1/1l* #3, and *Mpbmi1l-4* mutants. **j** MA plot showing differential gene expression (Log2 fold change (Log2fc)) in *Mpbmi1/1l* #1 mutants compared to WT. Significant gene expression changes are marked in red (log2 fold change ≥ 1 and adjusted *p* value ≤ 0.05) and blue (log2 fold change ≤ −1 and adjusted *p* value ≤ 0.05). **k**, **l** Boxplot showing the H2Aub (**k**) and H3K27me3 (**l**) levels (RPKM, reads per kilobase per million mapped reads) of upregulated and downregulated genes in *Mpbmi1/1l* #1 mutants. H2Aub and H3K27me3 levels were calculated as the average RPKM from 1 kb upstream of the transcriptional start to the transcriptional end of genes. Boxes show medians and the interquartile range, and error bars show the full range excluding outliers. **p < 0.01 (Wilcoxon test). **m** Percent of upregulated (Up) and downregulated (Down) genes marked by only-H2Aub, H2Aub/H3K27me3, and only-H3K27me3 in *Mpbmi1/1l* #1 mutants. **p < 0.01 (Hypergeometric test). **n** MA plot showing differential transposable element (TE) expression (Log2fc) in *Mpbmi1/1l* #1 mutants compared to WT. Significant TE expression changes are marked in red (log2 fold change ≥ 1 and adjusted *p* value ≤ 0.05) and blue (log2 fold change ≤ −1 and adjusted *p* value ≤ 0.05). **o** Percent of upregulated (Up_TE) and downregulated (Down_TE) TEs marked by only-H2Aub, H2Aub/H3K27me3, and only-H3K27me3 in *Mpbmi1/1l* #1 mutants. *p < 0.05; **p < 0.01 (Hypergeometric test)

In *Arabidopsis*, AtBMI1s have been shown to be involved in H2A ubiquitination [6, 20, 21, 33]. To explore the extent to which MpBMI1/1L function relies on H2Aub, we generated MpBMI1 knock out mutants by CRISPR/Cas9 in *Marchantia*. Using the AtBMI1A protein sequence as a query in a protein blast, we identified two genes (*Mp7g12670* and *Mp6g09730* in the MpTak1 v5.1 annotation) encoding AtBMI1 homologs in *Marchantia*. MpBMI1, encoded by *Mp7g12670*, contains an N-terminal RING finger domain and a C-terminal ubiquitin-like (RAWUL) domain [40], while *Mp6g09730* encodes for a protein named MpBMI1-LIKE (MpBMI1L) that only has a C-terminal RAWUL domain. The RAWUL domain is involved in protein-protein interaction and oligomerization of BMI1, which is essential for H2Aub activity of PRC1 in mammals [41–44]. We generated *Mpbmi1/1l* double knockout mutants by CRISPR/Cas9 and obtained combinations of double mutants with different mutations at the Cas9 target sites (mutant information shown in Additional file 1: Figures S7 and S8). Combinations of strong mutant alleles for both genes *Mpbmi1-1/Mpbmi1l-1* (named *Mpbmi1/1l*#1, Additional file 1: Figure S7a) and *Mpbmi1-2/Mpbmi1l-2* (named *Mpbmi1/1l* #2, Additional file 1: Figure S7b) caused strongly reduced growth rates and substantial size-reduction of gemma cups that contained only few and smaller gemmae compared to WT (Fig. 3a–c, f–h). The slightly more severe size reduction in *Mpbmi1/1l* #1 compared to *Mpbmi1/1l* #2 is likely due to the different extent of deletions and insertions caused by CRISPR/Cas9 in these two lines (Additional file 1: Figure S7). We also obtained one double mutant with a strong *Mpbmi1-3* allele and a weak *Mpbmi1l-3* allele (named *Mpbmi1/1l* #3, Additional file 1: Figure S8a) and one *Mpbmi1l-4* single mutant (Additional file 1: Figure S8b), which showed weakly reduced growth rates compared to WT (Fig. 3d, e). The fact that *Mpbmi1/1l* mutants had a more severe phenotype than *h2a_ub* mutants is consistent with the proposed redundant function of ubiquitination on K115/K116 and K119 in H2A. Western blot analysis revealed a global decrease of H2Aub in *Mpbmi1/1l* #1, *Mpbmi1/1l* #2, and *Mpbmi1/1l* #3 double mutants compared to WT (Fig. 3i), with a more pronounced reduction in the *Mpbmi1/1l* #1 mutant combination. We therefore used *Mpbmi1/1l* #1 in subsequent analyses. The residual H2Aub signal in the *Mpbmi1/1l* mutants possibly reflects remaining functional activity of MpBMI1/1L generated in the mutants. Alternatively, MpRING proteins have low functional activity in the absence of MpBMI1/1L. We found 2085 genes being upregulated and 1023 genes being downregulated in the *Mpbmi1/1l* mutants (Fig. 3j), suggesting that MpBMI1/1L mainly function as repressors, but also possibly as activators. We analyzed H2Aub levels on the deregulated genes in the *Mpbmi1/1l* mutants and found H2Aub to be significantly decreased on both upregulated genes and downregulated genes (Fig. 3k, Additional file 1: Figure S9a, b), implying that H2Aub is required for MpBMI1/1L-mediated gene silencing and activation. H3K27me3 level only decreased on upregulated but not on downregulated genes (Fig. 3l, Additional file 1: Figure S9c-f), indicating that PRC1-mediated gene silencing is dependent on H3K27me3. Accordingly, H2Aub marked genes were enriched in both upregulated and downregulated genes in *Mpbmi1/1l* mutants (Fig. 3m). There was also a large number of up- and downregulated TEs in *Mpbmi1/1l* mutants (Fig. 3n) that were enriched for H2Aub and H3K27me3 (Fig. 3o).

### Ubiquitination of H2AK115/K116 and H2AK119 is required for PRC1-mediated gene and TE expression

**Fig. 4:**
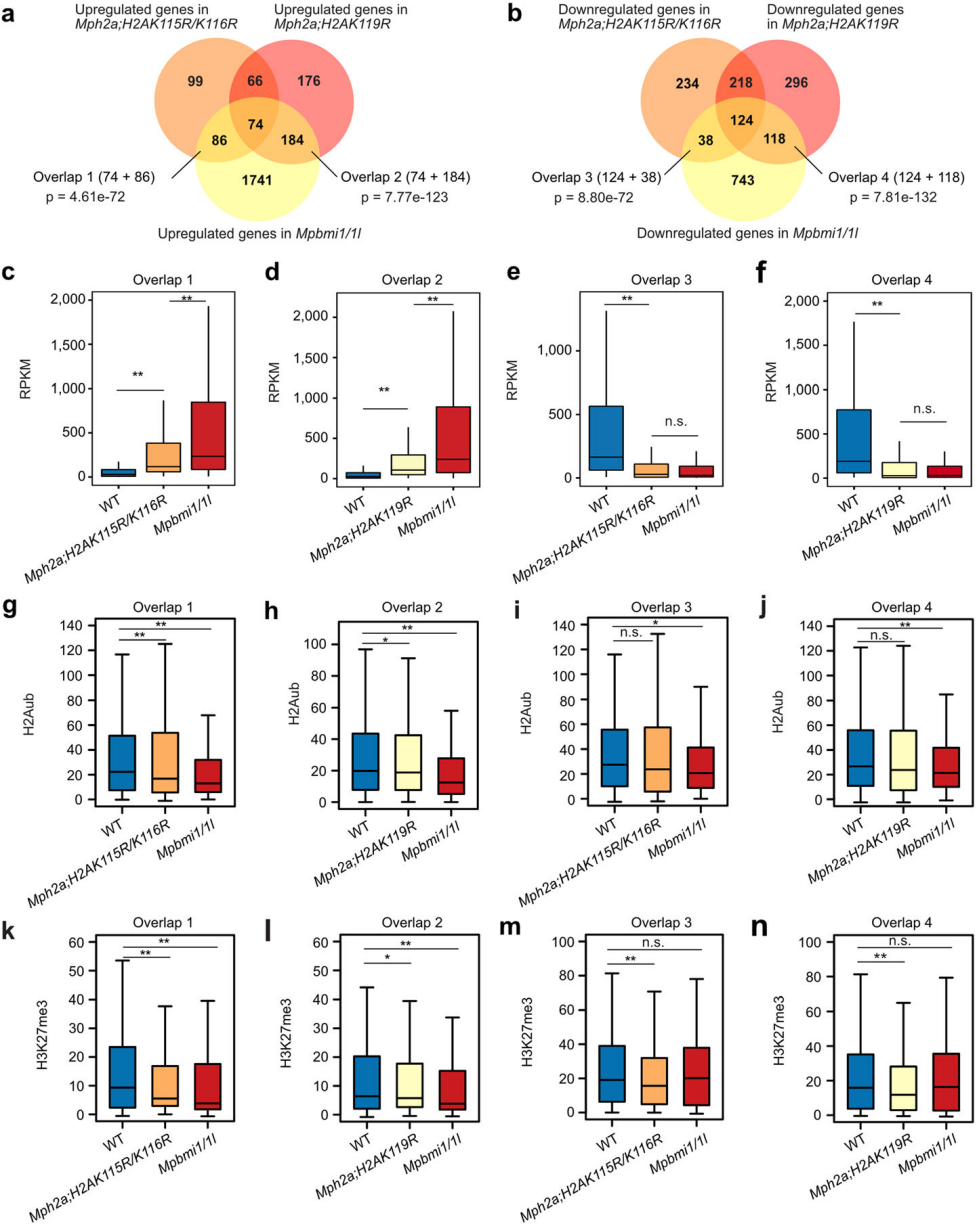
H2Aub contributes to PRC1-mediated transcriptional expression. **a** Venn diagram showing overlap of upregulated genes in *Mph2a;H2AK115R/K116R*, *Mph2a;H2AK119R*, and *Mpbmi1/1l* mutants. Significance was tested using a Hypergeometric test. **b** Venn diagram showing overlap of downregulated genes in *Mph2a;H2AK115R/K116R*, *Mph2a;H2AK119R*, and *Mpbmi1/1l* mutants. **c** Boxplot showing expression level (RPKM, reads per kilobase per million mapped reads) of genes in overlap 1 (panel a) in WT, *Mph2a;H2AK115R/K116R*, and *Mpbmi1/1l*. **d** Boxplot showing expression level (RPKM) of genes in overlap 2 (panel a) in WT, *Mph2a;H2AK119R*, and *Mpbmi1/1l*. **e** Boxplot showing expression level (RPKM) of genes in overlap 3 (panel b) in WT, *Mph2a;H2AK115R/K116R*, and *Mpbmi1/1l*. **f** Boxplot showing expression level (RPKM) of genes in overlap 4 (panel b) in WT, *Mph2a;H2AK119R* and *Mpbmi1/1l*. **g–j** Boxplots showing H2Aub levels of genes in the group of overlap 1 (**g**), overlap 2 (**h**), overlap 3 (**i**), and overlap 4 (**j**). H2Aub levels were calculated as the average RPKM from 1 kb upstream of the transcriptional start to the transcriptional start of genes. **k–n** Boxplots showing H3K27me3 levels of genes in the group of overlap 1 (**k**), overlap 2 (**l**), overlap 3 (**m**), and overlap 4 (**n**). H3K27me3 levels were calculated as the average RPKM from 1 kb upstream of the transcriptional start to the transcriptional start of genes. Boxes show medians and the interquartile range, and error bars show the full range excluding outliers. n.s., not significant; *p < 0.05, **p < 0.01 (Wilcoxon test)

**Fig. 5:**
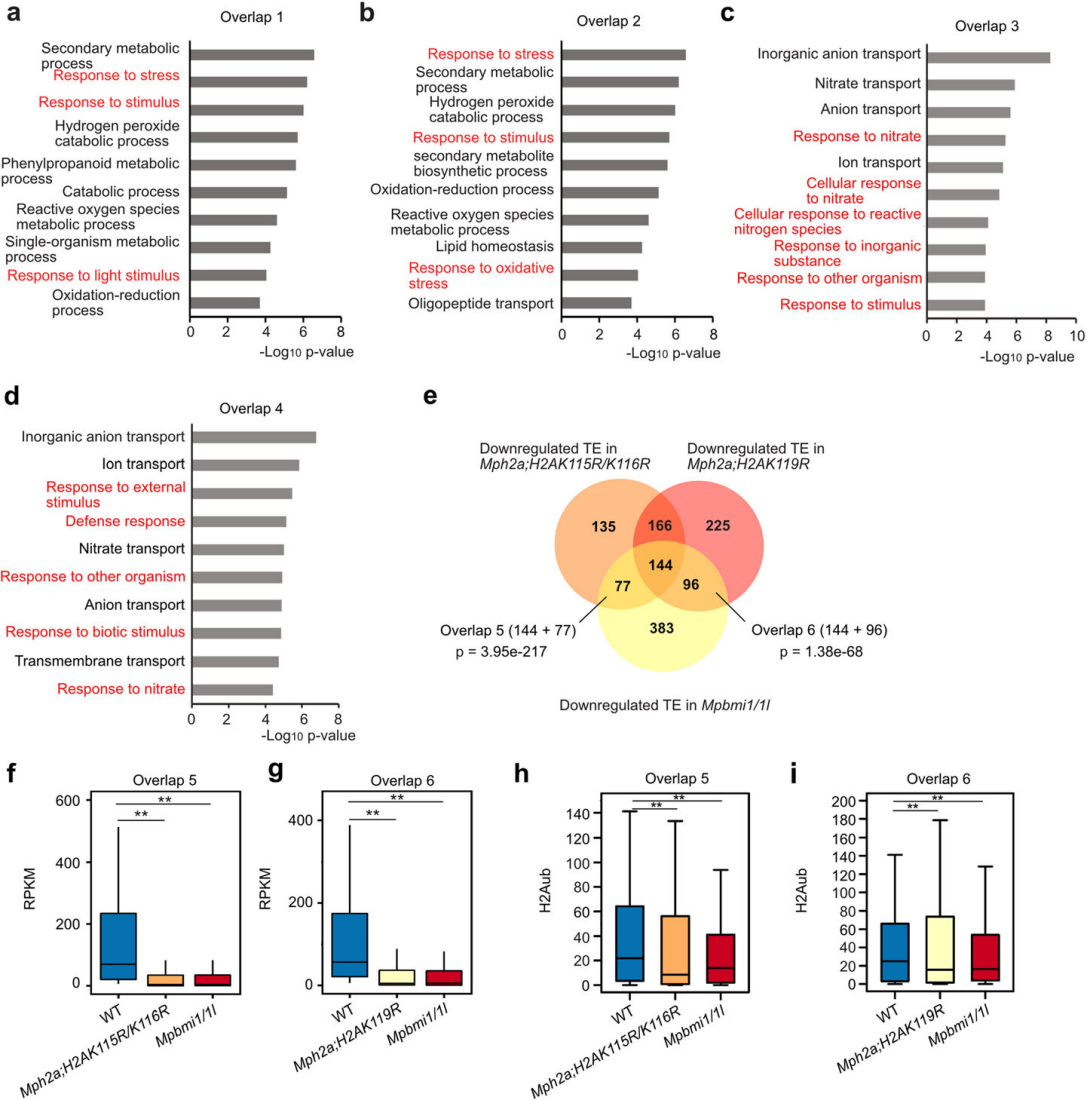
H2Aub is essential for PRC1-mediated transposable element activation. **a**, **b** Enriched GO terms of commonly upregulated genes in overlap 1 (panel **a** in Fig. 4) and overlap 2 (panel **a** in Fig. 4). Response pathways are marked in red. **c**, **d** Enriched GO terms of commonly downregulated genes in overlap 3 (panel **b** in Fig. 4) and overlap 4 (panel **b** in Fig. 4). Response pathways are marked in red. **e** Venn diagram showing overlap of downregulated transposable elements (TEs) in *Mph2a;H2AK115R/K116R*, *Mph2a;H2AK119R*, and *Mpbmi1/1l* mutants. Significance was tested using a Hypergeometric test. **f** Boxplot showing expression level (RPKM, reads per kilobase per million mapped reads) of TEs in overlap 5 (panel **e**) in WT, *Mph2a;H2AK115R/K116R*, and *Mpbmi1/1l* mutants. **g** Boxplot showing expression level (RPKM) of TEs in overlap 6 (panel **e**) in WT, *Mph2a;H2AK119R*, and *Mpbmi1/1l* mutants. **h**, **i** Boxplots showing H2Aub levels of TEs in the group of overlap 5 (**h**) and overlap 6 (**i**). H2Aub levels were calculated as the average RPKM from the start to the end of TEs. Boxes show medians and the interquartile range, and error bars show the full range excluding outliers. **p < 0.01 (Wilcoxon test)

To test whether impaired H2A ubiquitination and loss of PRC1 function has similar consequences, we compared transcriptome data of the *Mph2a;H2AK115R/K116R* and *Mph2a;H2AK119R* mutants with that of the *Mpbmi1/1l* mutants. Both *h2a_ub* mutants shared a significant number of upregulated genes (*p* = 2.36e−165) and we also found a significant overlap of upregulated genes between the *Mpbmi1/1l* mutants and *h2a_ub* mutants (Fig. 4a) as well as between all mutants (Fig. 4a, *p* = 3.37e−101). Similarly, a significant number of downregulated genes overlapped between two *h2a_ub* mutants and *Mpbmi1/1l* mutants (Fig. 4b). Genes commonly upregulated in *Mpbmi1/1l* and *Mph2a;H2AK115R/K116R* or *Mph2a;H2AK119R* mutants were more strongly upregulated in *Mpbmi1/1l* than in *h2a_ub* mutants (Fig. 4c, d), supporting the idea that mono-ubiquitination on H2AK115/K116 and H2AK119 is functionally redundant and mediated by MpBMI1/1L. Commonly downregulated genes in *h2a_ub* and *Mpbmi1/1l* mutants were expressed at similar low levels in *h2a_ub* and *Mpbmi1/1l* mutants (Fig. 4e, f), supporting the idea that H2Aub is required for PRC1-mediated gene activation. Consistent with the transcriptional state, H2Aub level was decreased in the promoter region of commonly upregulated genes in *h2a_ub* mutants and more strongly decreased with spanning into gene body in *Mpbmi1/1l* mutants (Fig. 4g, h, Additional file 1: Figure S10a, b), supporting the idea that PRC1-mediated H2Aub is required for gene repression. H2Aub was also significantly decreased in the promoter and gene body regions of downregulated genes in *Mpbmi1/1l* mutants (Fig. 4i, j, Additional file 1: Figure S10c, d), supporting an activating role of H2Aub. We noticed a trend of reduced H2Aub in the promoter region of downregulated genes in *h2a_ub* mutants, but the difference was not significant. This data suggests that in *h2a_ub* mutants, the ubiquitination of the non-mutated lysine residues H2AK115/K116 or H2AK119 is compensated, but is not functionally redundant for activating gene expression. Correlating with the H2Aub level, H3K27me3 was mildly decreased on the upregulated genes in *h2a_ub* mutants and strongly decreased in *Mpbmi1/1l* mutants (Fig. 4k, l, Additional file 1: Figure S10e, f). H3K27me3 was also decreased on the downregulated genes in *h2a_ub* mutants but not in *Mpbmi1/1l* mutants (Fig. 4m, n, Additional file 1: Figure S10g, h), indicating that PRC1-mediated gene activation is independent of H3K27me3. GO enrichment analyses of upregulated genes overlapping between *Mpbmi1/1l* and *Mph2a;H2AK115R/K116R* mutants (Fig. 5a) or *Mpbmi1/1l* and *Mph2a;H2AK119R* mutants (Fig. 5b) both showed that response pathways were over-represented. Among downregulated genes overlapped between *Mpbmi1/1l* and *h2a_ub* mutants, we also found a significant enrichment for response pathway-related GOs (Fig. 5c, d), which were nevertheless largely distinct from the enriched GO terms of upregulated genes.

Our data suggests that H2Aub is required for TE activation (Fig. 2o, p). Further supporting this idea, we also identified 700 downregulated TEs in *Mpbmi1/1l* mutants (Fig. 3n), of which a significant number overlapped with downregulated TEs in *h2a_ub* mutants (Fig. 5e). Like for genes, transcript levels of downregulated TEs were decreased to a similar level in *h2a_ub* and *Mpbmi1/1l* mutants (Fig 5f, g) and H2Aub levels were decreased even more strongly in *Mph2a;H2AK115R/K116R* and *Mph2a;H2AK119R* compared to *Mpbmi1/1l* mutants (Fig. 5h, i, and Additional file 1: Figure S11), suggesting that ubiquitination on H2AK115/K116 and H2AK119 is not functionally redundant for TE activation.

### H2Aub and H3K27me3 are affected in genes and TEs by the depletion of MpBMI1/1L

**Fig. 6:**
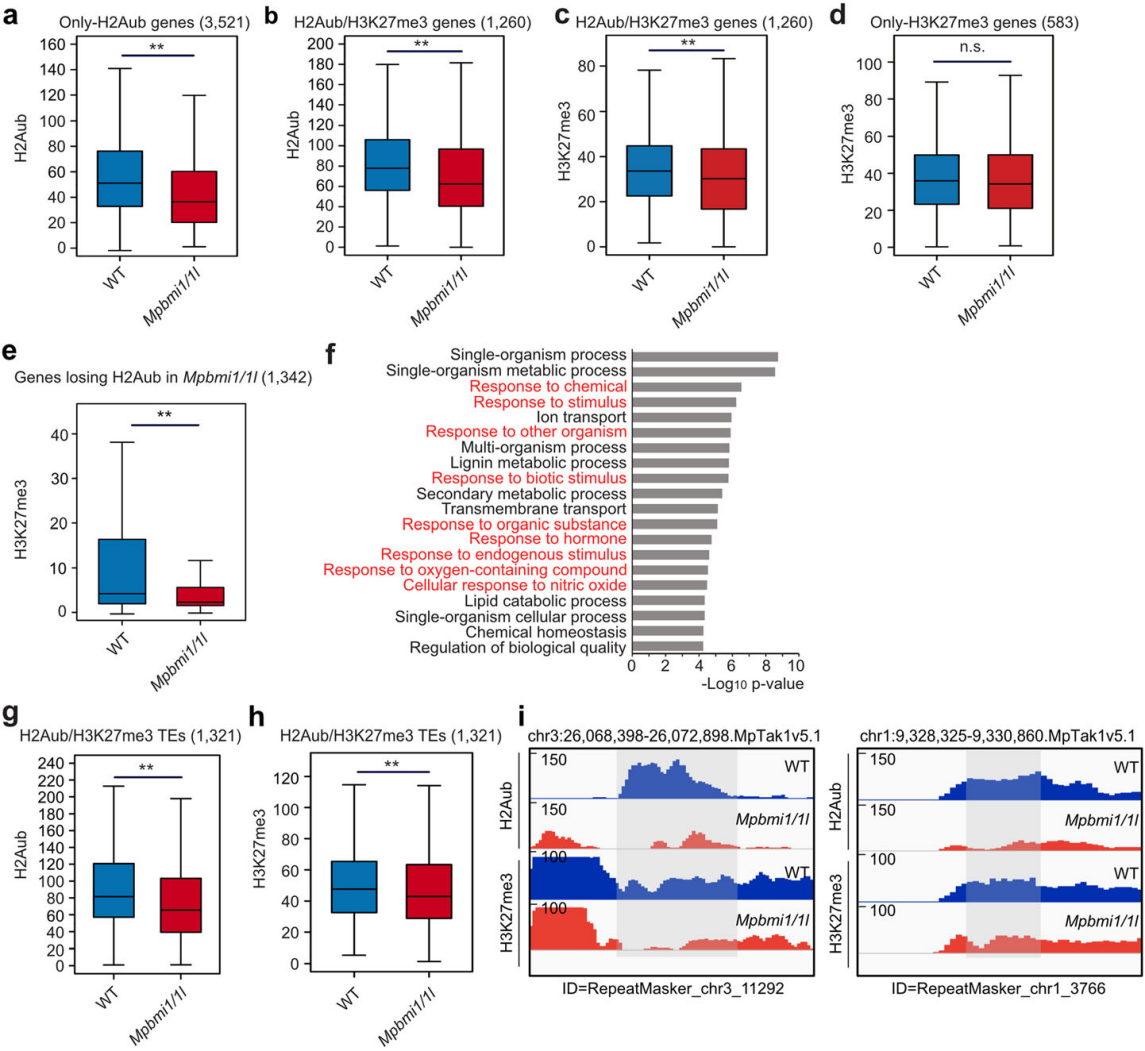
H3K27me3 is decreased on Polycomb target sites in genes and transposable elements (TEs) by depletion of MpBMI1/1L. **a**, **b** Boxplots showing H2Aub levels (RPKM, reads per kilobase per million mapped reads) on only-H2Aub genes (**a**) and H2Aub/H3K27me3 genes (**b**) in WT and *Mpbmi1/1l* mutants. **c**, **d** Boxplots showing H3K27me3 levels on H2Aub/H3K27me3 genes (**c**) and only-H3K27me3 genes (**d**) in WT and *Mpbmi1/1l* mutants. **e** Boxplot showing H3K27me3 levels of genes with reduced H2Aub level in *Mpbmi1/1l* mutants. H2Aub and H3K27me3 levels were calculated as the average RPKM from 1 kb upstream of the transcriptional start to the transcriptional end of genes. **f** GO terms of genes with reduced H2Aub level in *Mpbmi1/1l* mutants. Response pathways are marked in red. **g**, **h** Boxplots showing H2Aub (**g**) and H3K27me3 levels (**h**) on H2Aub/H3K27me3 TEs in wild type (WT) and *Mpbmi1/1l* mutants. H2Aub and H3K27me3 levels were calculated as the average RPKM from the start to the end of TEs. Boxes show medians and the interquartile range, and error bars show the full range excluding outliers. n.s., not significant; **p < 0.01 (Wilcoxon test). **i** Genome browser views of two selected H2Aub/H3K27me3 TE loci showing decreased H2Aub and H3K27me3 marks in *Mpbmi1/1l* mutants compared to WT

Consistent with the effect caused by AtBMI1 depletion in *Arabidopsis* [33], the H2Aub level was significantly decreased on only H2Aub genes as well as H2Aub/H3K27me3 genes in the *Mpbmi1/1l* mutants compared to WT (Fig. 6a, b, Additional file 1: Figure S12a, b). Also the H3K27me3 level was significantly decreased on H2Aub/H3K27me3 genes, yet not on only-H3K27me3 genes in the *Mpbmi1/1l* mutants compared to WT (Fig. 6c, d, Additional file 1: Figure S12c, d), implying that PRC1 activity mediates H3K27me3 deposition in *Marchantia*. The level H2Aub on only-H3K27me3 genes was unchanged in the *Mpbmi1/1l* mutants compared to WT (Additional file 1: Figure S12e, f), consistent with unchanged levels of H3K27me3. Conversely, genes losing H2Aub showed significantly reduced H3K27me3 levels in the *Mpbmi1/1l* mutants compared to WT (Fig. 6e). We found that genes losing H2Aub in *Mpbmi1/1l* mutants had lower H3K27me3 levels compared to H2Aub/H3K27me3 and H3K27me3 only genes (Fig. 6c, d); nevertheless, a significant proportion of genes losing H2Aub in *Mpbmi1/1l* or *h2a_ub* mutants are marked by H3K27me3 (Additional file 1: Figure S12g-i). We tested whether genes losing H2Aub in the *Mpbmi1/1l* mutants belonged to specific pathways. Among the top twenty significantly enriched GO terms, nine GO terms corresponded to multiple response pathways to intrinsic and extrinsic stimuli (Fig. 6f), which occurred in the commonly upregulated and downregulated genes between *Mpbmi1/1l* and *h2a_ub* mutants. We tested whether the connection between H2Aub and H3K27me3 was restricted to genes or was also present in TEs. Loss of MpBMI1/1L caused a significant decrease of both H2Aub and H3K27me3 levels on H2Aub/H3K27me3 TEs (Fig. 6g–i), revealing that PRC1-mediated recruitment of PRC2 is not restricted to genic regions.

## Discussion

Understanding the extent to which the function of histone modifying enzymes requires their catalytic activity is an ongoing challenge in the chromatin field. While recent work revealed that the catalytic activity of PRC1 is required in mouse ESCs [28, 29], whether this requirement is evolutionary conserved remains to be demonstrated. We found that PRC1-catalyzed H2Aub contributes to the Polycomb-mediated transcriptional repression in *Marchantia*, similar to the reported requirements in mouse ESCs [28, 29]. Our study thus supports an evolutionarily conserved Polycomb mechanism in plants and animals.

Loss of MpBMI1/1L activity in *Marchantia* impaired H3K27me3 deposition on H2Aub and H3K27me3 marked genes, similar to reported findings in *Arabidopsis* [33]. Nevertheless, it was previously unknown whether the reduction of H3K27me3 in *Atbmi1* in *Arabidopsis* is a consequence of decreased PRC1 catalytic activity or PRC1 non-catalytic activity, since PRC1 and PRC2 were shown to interact [19, 20, 36]. By comparing the H2Aub deficient mutants *Mph2a;H2AK115R/K116*R and *Mph2a;H2AK119R* with *Mpbmi1/1l* mutants, we discovered that reduction of H3K27me3 levels on Polycomb target genes in *Mpbmi1/1l* mutants also occurred in *h2a_ub* mutants that evade ubiquitination, demonstrating that H2Aub directly affects H3K27me3 deposition. Previous work showed that PRC1 initiates silencing, followed by PRC2-mediated H3K27me3 that maintains stable repression in *Arabidopsis* [6, 21, 33, 34]. Our data adds support to this model and extends it by showing that the PRC1-mediated H2Aub is essential for the initial PRC2-mediated repression.

In *Marchantia*, H3K27me3 is located in heterochromatic regions and marks TEs and repeats [39], contrasting its mainly genic localization in *Arabidopsis* [33]. We showed that PRC1-catalyzed H2Aub is required for TE activation in *Marchantia*, revealing a new role of PRC1 beyond the Polycomb regulatory network. TE activation frequently correlates with loss of DNA methylation in *Arabidopsis* [45]. Whether H2Aub has antagonizing roles with DNA methylation in *Marchantia* remains to be explored.

The failure to obtain *Mph2a;H2AK115R/K116R/K119R* mutants with complete loss of H2Aub strongly suggests that H2Aub has essential functions in *Marchantia*. Similarly, H2Aub-deficient *Drosophila* embryos arrest at the end of embryogenesis, indicating that the requirement of H2Aub to regulate essential biological functions is evolutionary conserved [30]. As for H2Aub deficiency, also loss of the RING1 encoding gene *Sce* causes arrest of embryo development in *Drosophila* [30]. In contrast, we found that mutants in *MpBMI1/1L* are viable and similarly, mutants in *Arabidopsis* BMI encoding genes are also viable [21]. Nevertheless, it is possible that in both systems BMI function is not completely depleted, since the *Atbmi1b* and *Atbmi1c* mutant alleles are probably not complete null alleles in *Arabidopsis* [6, 20] and we found remaining H2Aub present in *Mpbmi1/l* mutants (Fig. 3i), pointing that the alleles have residual activity. We failed to obtain CRISPR/Cas9 mutants using guide RNAs targeting an N-terminal region in the MpBMI1, suggesting that complete loss of PRC1 function is lethal. Nevertheless, it is also possible that RING proteins can have catalytic activity independently of BMI proteins, as suggested based on in vitro catalytic activity of AtRING1A and AtRING1B proteins in *Arabidopsis* [6]. Previous work revealed that the H2A variant H2A.Z can be ubiquitinated in *Arabidopsis* and incorporation of this modification is required for H2A.Z-mediated transcriptional repression [46]. It is possible that MpBMI1/1L also affects ubiquitination of the H2A variant H2A.Z, which could provide an alternative explanation for the more severe phenotype of *Mpbmi1/1l* mutants compared to *h2a_ub.* Due to the lack of a suitable antibody, this possibility could not be tested.

Previous work revealed that PRC1-mediated H2Aub is associated with gene responsiveness and that responsive genes require H2Aub to initiate PRC2-mediated repression in *Arabidopsis* [34, 35]. At the same time, for stable gene repression H2Aub needs to be removed by the H2A deubiquitinases UBP12 and UBP13, likely because the occurrence of H2Aub allows recruitment of the H3K27me3 demethylase REF6 in *Arabidopsis* [34]. The association of H2Aub with gene activation is also supported by our study; nevertheless, the fact that we found reduced H3K27me3 levels on downregulated genes in *h2a_ub* mutants suggests that H2Aub also has a direct role for gene activation independent of H3K27me3.

A significant number of downregulated genes overlapped between *h2a_ub* and *Mpbmi1/1l* mutants, suggesting that the catalytic activity of PRC1 is required for PRC1-mediated gene activation. It was proposed that the PRC1-catalytic activity may be dispensable for PRC1 function in promoting the expression of active genes in mammalian systems [47]; however, our data rather suggests that PRC1-catalyzed H2Aub is required for gene activation.

## Conclusions

In summary, we show that the ubiquitinated lysines in MpH2A act redundantly and H2Aub directly contributes to the deposition of H3K27me3 in *Marchantia*, demonstrating the determinant role of PRC1 catalysis in the Polycomb repressive system. Together with previous findings in *Arabidopsis* and mouse ESCs [28, 29, 33], our study supports an evolutionarily conserved Polycomb mechanism in divergent land plants and animals. Our finding strongly supports a model in which the catalytic activity of PRC1 is required for PRC2-mediated gene repression and at the same time required for PRC2-independent gene activation.

## Methods

### Plant material and growth conditions

*Marchantia polymorpha* ssp. ruderalis Uppsala accession (Upp) was used as WT and for transformation [48]. Plants were grown on vented petri dishes containing Gamborg’s B5 medium solidified with 1.4% plant agar, pH 5.5, under 16/8 h photoperiod at 22°C with a light intensity of 60–70 umol m^−2^ s^−1^. Plate lids were taped to prevent loss of water.

### Generation of DNA constructs

Vectors pMpGE_En03, pMpGE010, pMpGWB401, and pMpGWB403 used in this study were previously described [49, 50]. DNA fragments used to generate the guide RNAs against *MpBMI1* and *MpBMI1L* were prepared by annealing two pairs of primers (LH4513/LH4514, LH4517/LH4518, specified in Additional file 2: Table S1). The fragments were inserted into the BsaI site of pMpGE_En03 to yield pMpGE_En03-MpBMI1gRNA02 and pMpGE_En03-MpBMI1LgRNA04, respectively, and then transferred into pMpGE010 and pMpGWB401 using the Gateway LR reaction (Thermo Fisher Scientific) to generate pMpGE010_MpBMI1gRNA and pMpGWB401_MpBMI1LgRNA. Similarly, the two pairs of primers (LH4012/LH4013, LH4303/LH4304) were used to generate pMpGE_En03-MpH2AgRNA2 and pMpGE_En03-MpH2AgRNA3, which were subsequently transferred into pMpGE010 to generate pMpGE010-MpH2AgRNA2 and pMpGE010-MpH2AgRNA3, respectively. H2AK115R/K116R and H2AK119R were amplified by two pairs of primers (LH3848/LH4309 and LH3848/LH3849) and sub-cloned into the pENTR-TOPO vector (Thermo Fisher Scientific) to generate pENTR-H2AK115R/K116R and pENTR-H2AK119R, respectively. The pENTR vectors were transferred into pMpGWB403 by Gateway LR reaction to yield pMpGWB403-H2AK115R/K115R and pMpGWB403-H2AK119R. Primers used are listed in Additional file 2: Table S1.

### Generation of transgenic *Marchantia polymorpha*

The constructs were transformed into spores of *Marchantia* by Agrobacterium GV3101 as described previously [51]. Spores were grown in liquid Gamborg’s B5 medium with 2% sucrose, 0.1% Casamino acids, and 0.03% L-Glutamine for 10 days under constant light. Agrobacteria containing constructs were grown in liquid LB with antibiotic for 2 days and then pelleted. The pellet was resuspended in the spore growth media with 100 mM acetosyringone and grown for 4 h at 28°C with spinning. Agrobacteria suspension was added to spores together with acetosyringone to a final concentration of 100 mM and the mixture was grown for another 2 days. Sporelings were plated on selection media with 200 mg/ml Timentin. Several independent primary transformants (T1 generation) were analyzed for the presence of the transgene by genomic PCR.

### Antibodies

The antibodies used were anti-H3 (07-690, Merck Millipore, Burlington, MA, USA), anti-H2Aub (#8240S, Cell signalling technology, Danvers, MA), and anti-H3K27me3 (07-449, Merck Millipore).

### Histone extraction and western blotting

Histone extraction and western blotting of 15-day-old seedlings were performed as previously described [36]. Fully uncropped western blots can be found in Additional file 1: Figure S13.

### RNA sequencing

Fifty milligrams of 15-day-old thalli of WT, *Mpbmi1/1l*, *Mph2a;H2AK115R/K116R*, and *Mph2a;H2AK119R* mutants were used for RNA extraction. RNA was extracted using the MagMAX™ Plant RNA Isolation Kit (Thermo Fisher Scientific) in biological triplicates. Libraries were generated using DNA-free RNA with the NEBNext® Ultra™ II RNA Library Prep Kit for Illumina according to manufacturer’s instructions. Sequencing was performed on an Illumina HiSeq2000 in 150-bp pair-end mode at Novogene (Hong Kong).

### Transcriptome data analysis

Untrimmed reads were mapped to the *Marchantia polymorpha* MpTak1 v5.1 reference genome [39] using STAR (v2.5.3.a [52]). Read numbers of mapping statistics are reported in Additional file 3: Table S2. Expression counts were generated using the R function summarizeOverlaps from the package HTSeq in union mode on exons from the reference transcriptome MpTak1v5.1_r1. A comparison of RPKM in RNA-seq triplicates showed high reproducibility of data in Additional file 1: Figure S14. Differential expression analyses were performed using the R package DESeq2 (v1.20.0 [53]). Genes or TEs with an absolute log2 fold change ≥ 1 and FDR ≤ 0.05 were considered as differentially expressed. Differential expressed genes in *h2a_ub* and *Mpbmi1/1l* mutants can be found in Additional file 4: Table S3. Differential expressed TEs in *h2a_ub* and *Mpbmi1/1l* mutants can be found in Additional file 5: Table S4.

### H3, H2Aub1, and H3K27me3 ChIP-seq

For H3, H2Aub, and H3K27me3 ChIP-seq, WT, *Mpbmi1/1l*, *Mph2a;H2AK115R/K116R*, and *Mph2a;H2AK119R* plants were grown for 15 days on B5 medium, and then about 300 mg thalli were harvested. ChIP was performed as described before [54]. In short, vacuum infiltration with formaldehyde was performed for 2 × 10 min. Crosslinking was quenched by adding glycine to a final concentration of 0.125 M under another 5-min vacuum infiltration. Sonication of the chromatin was done for eight 30-s ON, 30-s OFF cycles. Overnight antibody binding was performed directly after sonication, followed by adding washed protein A dynabeads (Thermo Fisher Scientific) to each ChIP aliquot. De-crosslinking and subsequent DNA recovery steps were performed using the Ipure kit v2 (Diagenode, Liège, Belgium). The Ovation Ultralow Sytem V2 (NuGEN, Redwood city, CA, USA) was used for the ChIP-seq library preparation, and 150-bp paired-end sequencing was performed on the HiseqX platform at Novogene (Hong Kong). The ChIP-seq experiments were done using two biological replicates per IP, per genotype.

### Quality control and read mapping for ChIP-seq

FastQC (https://www.bioinformatics.babraham.ac.uk/projects/fastqc/) was used to examine read quality of each sample. Low quality ends (phred < 20) and adapter sequences were removed with Trimmomatic (v0.39 [55]). Reads with low average quality were also discarded (phred < 28). For all experiments, reads were mapped to the *M. polymorpha* reference genome MpTak1 v5.1 using bowtie2 (v2.3.5.1 [56]). Details on read numbers can be found in Additional file 6: Table S5. Genome sequence and gene annotation data were downloaded from the *Marchantia* website (marchantia.info).

### Peak calling

H2Aub and H3K27me3 aligned .sam files were imported into Homer [57]. Duplicated mappings were removed using Homer. Peak calling was done using Homer with histone style settings and using histone H3 as the background to control for nucleosome occupancy. The same analysis was performed with the published WT H3K27me3 data (SRA: PRJNA553138) in *Marchantia* [39]. The peak tag counts were generated by Homer. A comparison of normalized peak tag counts (RPKM) by Homer in ChIP-seq replicates showed high reproducibility of data in Additional file 1: Figure S15. Only peaks present in two replicates of ChIP-seq data were considered as real peaks and retained for subsequent analyses. Peaks were correlated with a gene when the peaks were located at any region of this gene or at most 2 kb upstream of its transcription start site. Lists of genes defined by the presence of H2Aub and H3K27me3 are shown in Additional file 7: Table S6. TEs covered by H2Aub and H3K27me3 are listed in Additional file 8: Table S7. The statistical comparison of differential peak tag counts was performed with DEseq2 package in R using the raw tag counts outputs from Homer. Peaks with the adjusted *p*-value (FDR) < 0.05 were considered as differentially changed peaks. Differential H2Aub peaks in *h2a_ub* and *Mpbmi1/1l* mutants can be found in Additional file 9: Table S8. Differential H3K27me3 peaks in *h2a_ub* and *Mpbmi1/1l* mutants can be found in Additional file 10: Table S9.

### Peak visualization

Peak profiles were visualized by the Integrative Genome Viewer (IGV) [58]. Bigwig files were outputted from “bamCoverage” function in deepTools [59] using Reads Per Kilobase Million (RPKM) as normalization parameter. The Bigwig files were further used in the “computMatrix” function in deepTools with the “scale-regions” as setting parameter to generate H2Aub and H3K27me3 matrix on genes from 3 kb upstream of the transcriptional start to 3 kb downstream of the transcriptional end of genes and on TEs from 3 kb upstream of the start to 3 kb downstream of the end of TEs with a bin size of 50 bp. H2Aub and H3K27me3 levels (RPKM) on genes and TEs in boxplots were calculated as the average RPKM on defined regions for specified group of genes and TEs.

### GO analysis

GO analysis was performed on the *Arabidopsis* homologs of *Marchantia* genes. The homologs of *Marchantia* genes in *Arabidopsis* were retrieved from PLAZA 4.0 DICOT, inferred by the Best-Hits-and-Inparalogs (BHIF) approach ([60], https://bioinformatics.psb.ugent.be/plaza/versions/plaza_v4_dicots/, Additional file 11: Table S10). GO term enrichment was performed in PLAZA 4.0 DICOT.

## Supplementary Information

**Additional file 1: Figure S1.** Strategy of generating H2Aub depleted lines. **Figure S2.** Comparison of H3K27me3 data of this study with previously published data. **Figure S3.** H2Aub is essential for H3K27me3 incorporation. **Figure S4.** Distribution of H2Aub and H3K27me3 peaks among functional features of the *Marchantia* genome. **Figure S5.** H3K27me3 targeting is generally affected on genes in H2Aub deficient lines. **Figure S6.** H2Aub is required for gene repression and activation. **Figure S7.** Characterization of strong double mutant allele combinations in *MpBMI1/1L.* **Figure S8.** Characterization of weak double mutant allele combinations in *MpBMI1/1L.* **Figure S9.** Deposition of H2Aub is affected on upregulated and downregulated genes by loss of MpBMI1/1L. **Figure S10.** PRC1-mediated gene repression and activation depends on H2Aub. **Figure S11.** H2Aub contributes to PRC1-mediated transposable element activation. **Figure S12.** H2Aub and H3K27me3 deposition are affected by loss of MpBMI1/1L. **Figure S13.** Western blots of H2A and H2Aub**. Figure S14.** Scatter plots comparing RNA-seq triplicates. **Figure S15.** Scatter plots comparing ChIP-seq replicates.

**Additional file 2: Table S1.** Primers used in the constructs.

**Additional file 3: Table S2.** Mapping statistics of RNA-seq data.

**Additional file 4: Table S3.** Genes deregulated in *h2a_ub* and *Mpbmi1/1l *mutants.

**Additional file 5: Table S4.** TEs deregulated in *h2a_ub* and *Mpbmi1/1l* mutants.

**Additional file 6: Table S5.** Mapping statistics of ChIP-seq data.

**Additional file 7: Table S6.** Three specified group of genes defined by H2Aub and H3K27me3 marks.

**Additional file 8: Table S7.** TEs marked by H2Aub and H3K27me3.

**Additional file 9: Table S8.** Differential H2Aub peaks in *h2a_ub* and *Mpbmi1/1l* mutants.

**Additional file 10: Table S9.** Differential H3K27me3 peaks in *h2a_ub* and *Mpbmi1/1l* mutants.

**Additional file 11: Table S10.** Arabidopsis homologs of Marchantia genes.

**Additional file 12.** Review history.

## Data Availability

The datasets supporting the conclusions of this article are available in the Gene Expression Omnibus (GEO) with the accession number GSE164394 [61].
