# Supplementary material for: H2A ubiquitination is essential for Polycomb Repressive Complex 1-mediated gene regulation in Marchantia polymorpha
Source: Genome Biol. 2021 Sep 1;22:253. doi: 10.1186/s13059-021-02476-y (PMC8408974; doi:10.1186/s13059-021-02476-y)
Supplement: Supplementary file 1 — Additional file 1: Figure S1. Strategy of generating H2Aub depleted lines. Figure S2. Comparison of H3K27me3 data of this study with previously published data. Figure S3. H2Aub is essential for H3K27me3 incorporation. Figure S4. Distribution of H2Aub and H3K27me3 peaks among functional features of the Marchantia genome. Figure S5. H3K27me3 targeting is generally affected on genes in H2Aub deficient lines. Figure S6. H2Aub is required for gene repression and activation. Figure S7. Characterization of strong double mutant allele combinations in MpBMI1/1L. Figure S8. Characterization of weak double mutant allele combinations in MpBMI1/1L. Figure S9. Deposition of H2Aub is affected on upregulated and downregulated genes by loss of MpBMI1/1L. Figure S10. PRC1-mediated gene repression and activation depends on H2Aub. Figure S11. H2Aub contributes to PRC1-mediated transposable element activation. Figure S12. H2Aub and H3K27me3 deposition are affected by loss of MpBMI1/1L. Figure S13. Western blots of H2A and H2Aub. Figure S14. Scatter plots comparing RNA-seq triplicates. Figure S15. Scatter plots comparing ChIP-seq replicates. [file 13059_2021_2476_MOESM1_ESM.docx]

**
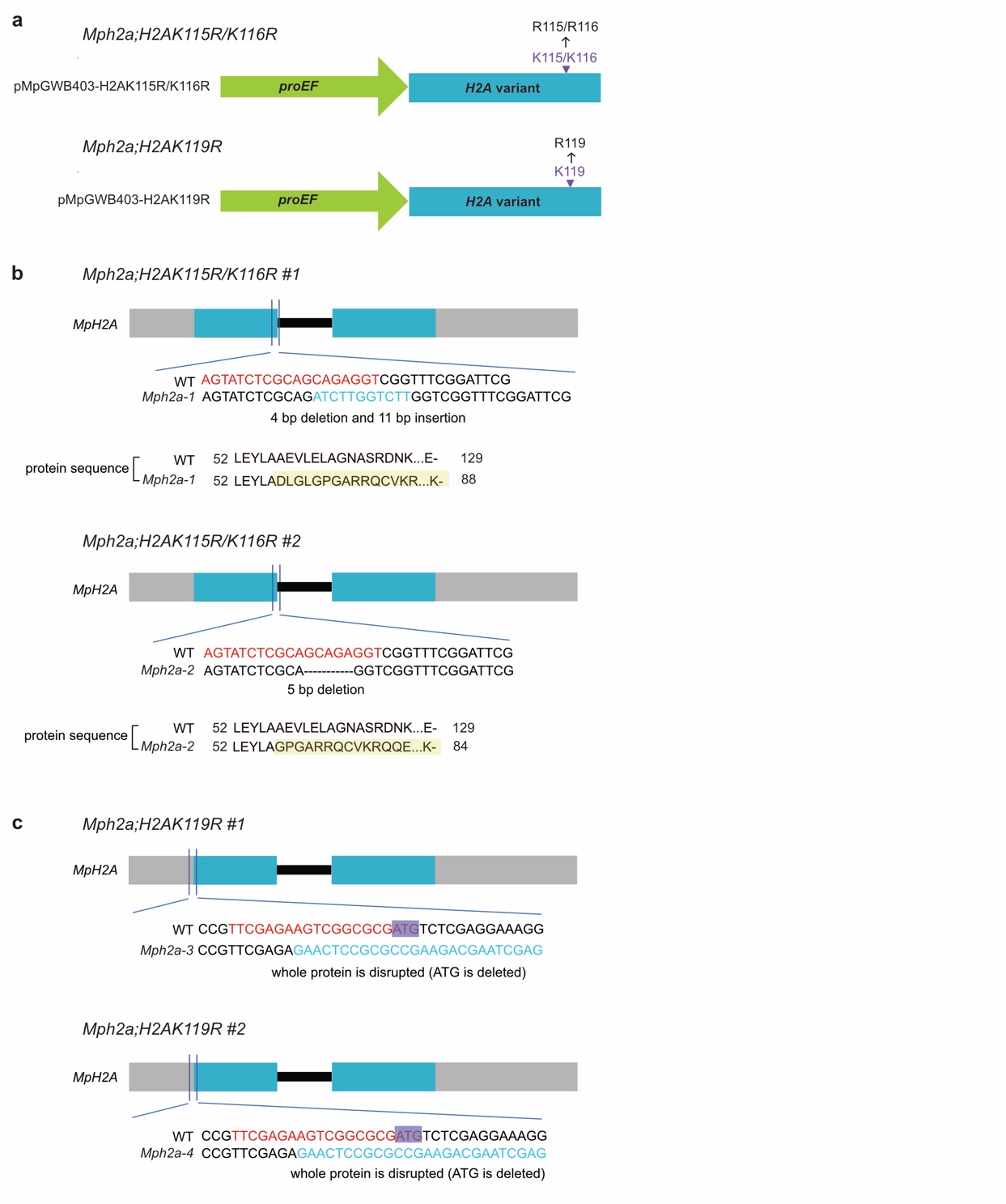
**

**Figure S1 Strategy of generating H2Aub depleted lines.**

***a.*** *H2A* variants driven by *MpEF* promoter (pMpGWB403-H2AK115R/K116R and pMpGWB403-H2AK119R) and CRISPR/Cas9 constructs with target gRNAs (panel b, c) were co-transformed into *Marchantia* to generate *Mph2a;H2AK115R/K116R* and *Mph2a;H2AK119R* plants. **b, c.** Disruption of the endogenous *MpH2A* by CRISPR/Cas9. Nucleotide and protein sequence alignments of wild-type (WT) and mutant alleles of MpH2A. *Mph2a;H2AK115R/K116R* #1 refers to *Mph2a-1* expressing *H2AK115R/K116R*, *Mph2a;H2AK115R/K116R* #2 refers to *Mph2a-2* expressing *H2AK115R/K116R*, *Mph2a;H2AK119R* #1 refers to *Mph2a-3* expressing *H2AK119R,* and *Mph2a;H2AK119R* #2 refers to *Mph2a-4* expressing *H2AK119R*. gRNA sequences are shown in red. Newly inserted nucleotides are shown in light blue. Nonsense protein sequences are shaded in yellow. “-” refers to the stop codon. ATG in purple shadow refers to the start codon.


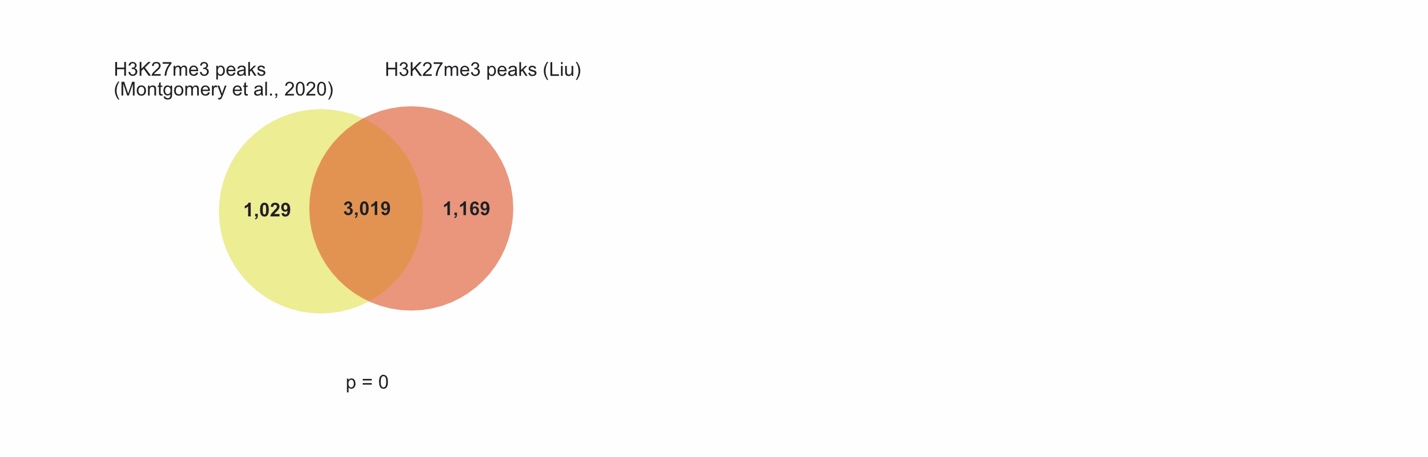


**Figure S2 Comparison of H3K27me3 data of this study with previously published data.**

Venn diagram showing overlap of wild-type H3K27me3 peaks generated in this study with previously published H3K27me3 peaks (Montgomery et al. 2020). Significance was tested using a Hypergeometric test.


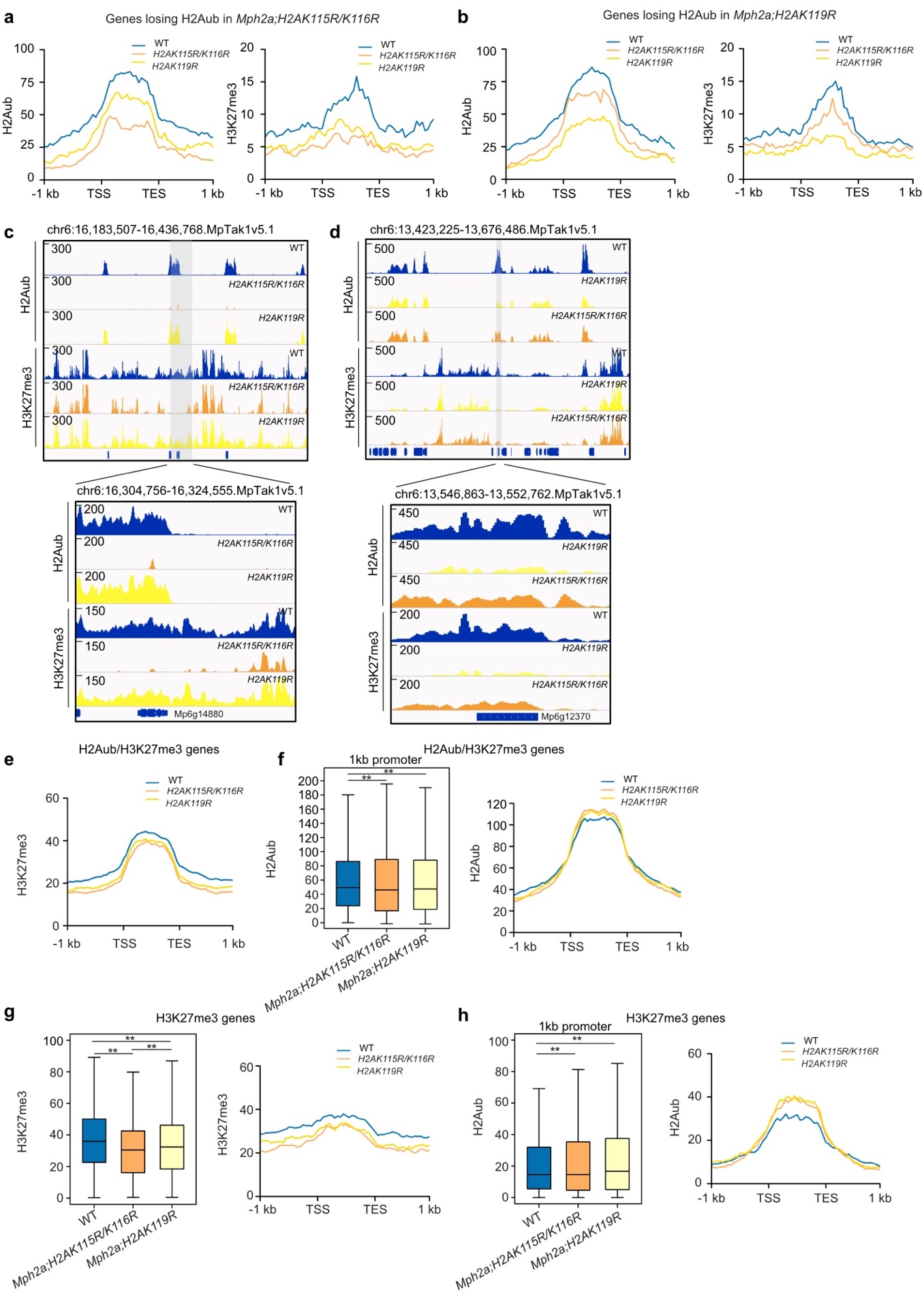


**Figure S3. H2Aub is essential for H3K27me3 incorporation.**

**a.** Metagene plot showing median values of H2Aub and H3K27me3 levels (RPKM, reads per kilobase per million mapped reads) of genes losing H2Aub in *Mph2a;H2AK115R/K116R* in WT, *Mph2a;H2AK115R/K116R* (*H2AK115R/K116R*) and *Mph2a;H2AK119R* (*H2AK119R*) mutants. **b.** Metagene plot showing median values of H2Aub and H3K27me3 levels (RPKM) of genes losing H2Aub in *Mph2a;H2AK119R* in WT, *Mph2a;H2AK119R* and *Mph2a;H2AK115R/K116R* mutants. **c.** Genomic snapshot of a gene with reduced H2Aub level in *Mph2a;H2AK115R/K116R* mutant. Lower panel shows a zoom in of the region marked in gray in the upper panel. **d.** Genomic snapshot of a gene with reduced H2Aub level in *Mph2a;H2AK119R* mutants. Lower panel shows a zoom in of the region marked in gray in the upper panel. **e.** Metagene plot showing H3K27me3 levels of H2Aub/H3K27me3 genes in WT, *Mph2a;H2AK115R/K116R* and *Mph2a;H2AK119R* mutants. **f.** Boxplot and metagene plot showing H2Aub levels of only-H3K27me3 genes in WT, *Mph2a;H2AK115R/K116R* and *Mph2a;H2AK119R* mutants. H2Aub levels in boxplot were calculated as the average RPKM from 1 kb upstream of the transcriptional start to the transcriptional start of genes. **g.** Boxplot and metagene plot showing H3K27me3 levels on only-H3K27me3 genes in WT, *Mph2a;H2AK115R/K116R* and *Mph2a;H2AK119R* mutants. H3K27me3 levels were calculated as the average RPKM from 1 kb upstream of the transcriptional start to the transcriptional end of genes. **h.** Boxplot and metagene plot showing H2Aub levels (RPKM) on only-H3K27me3 genes in WT, *Mph2a;H2AK115R/K116R* and *Mph2a;H2AK119R* mutants. H2Aub levels were calculated as the average RPKM from 1 kb upstream of the transcriptional start to the transcriptional start of genes. Boxes show medians and the interquartile range, and error bars show the full range excluding outliers. **, p < 0.01 (Wilcoxon test).


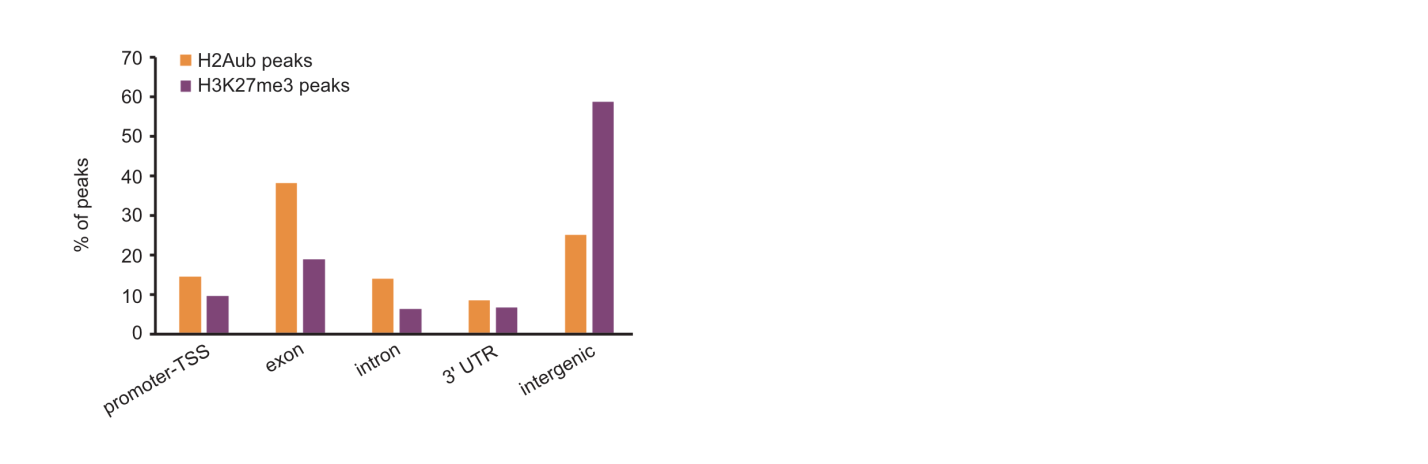


**Figure S4 Distribution of H2Aub and H3K27me3 peaks among functional features of the *Marchantia* genome.**


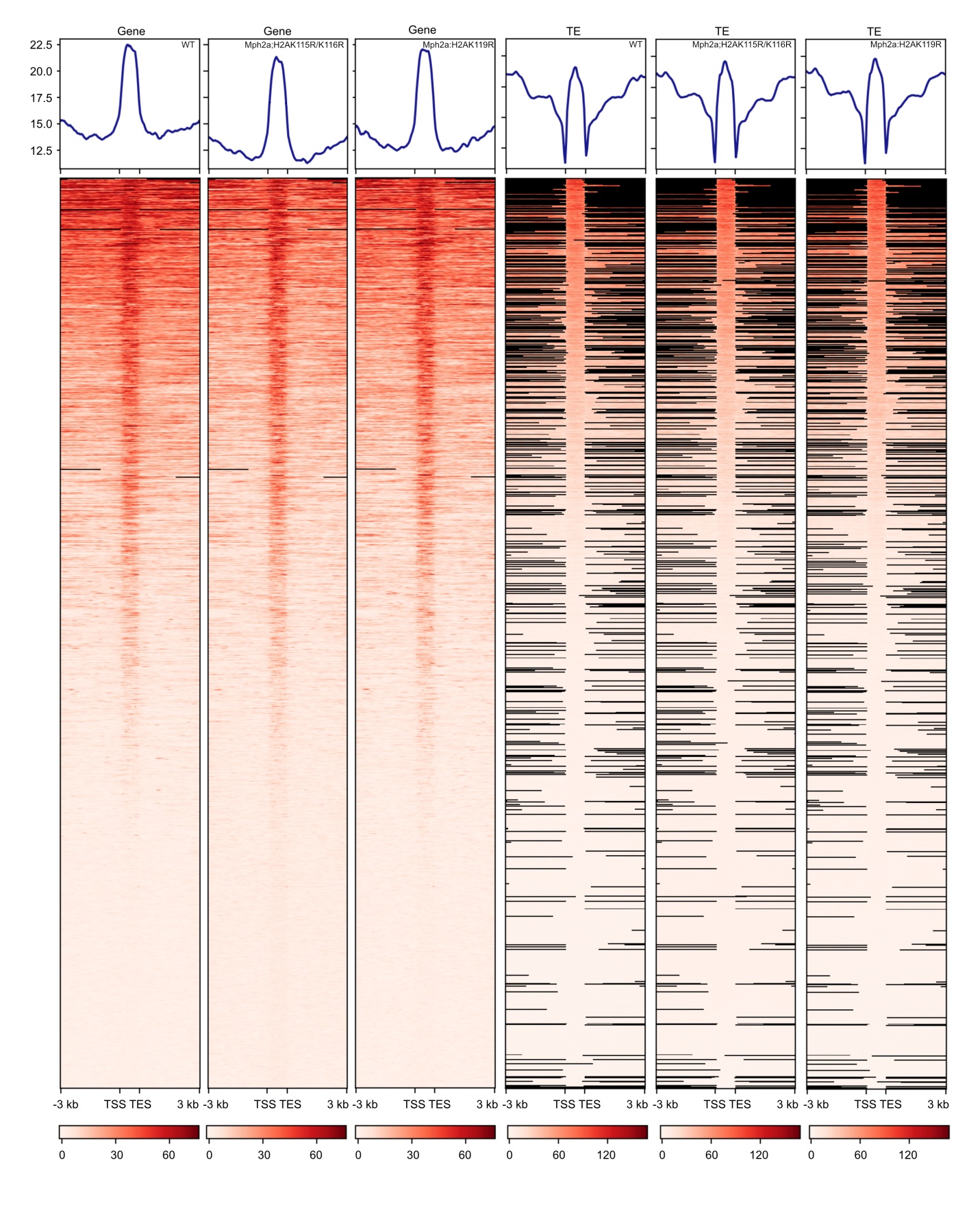


**Figure S5 H3K27me3 targeting is generally affected on genes in H2Aub deficient lines.**

Metagene plots and heatmaps showing H3K27me3 levels (RPKM, reads per kilobase per million mapped reads) on all genes and TEs in WT, *Mph2a;H2AK115R/K116R* and *Mph2a;H2AK119R* mutants.


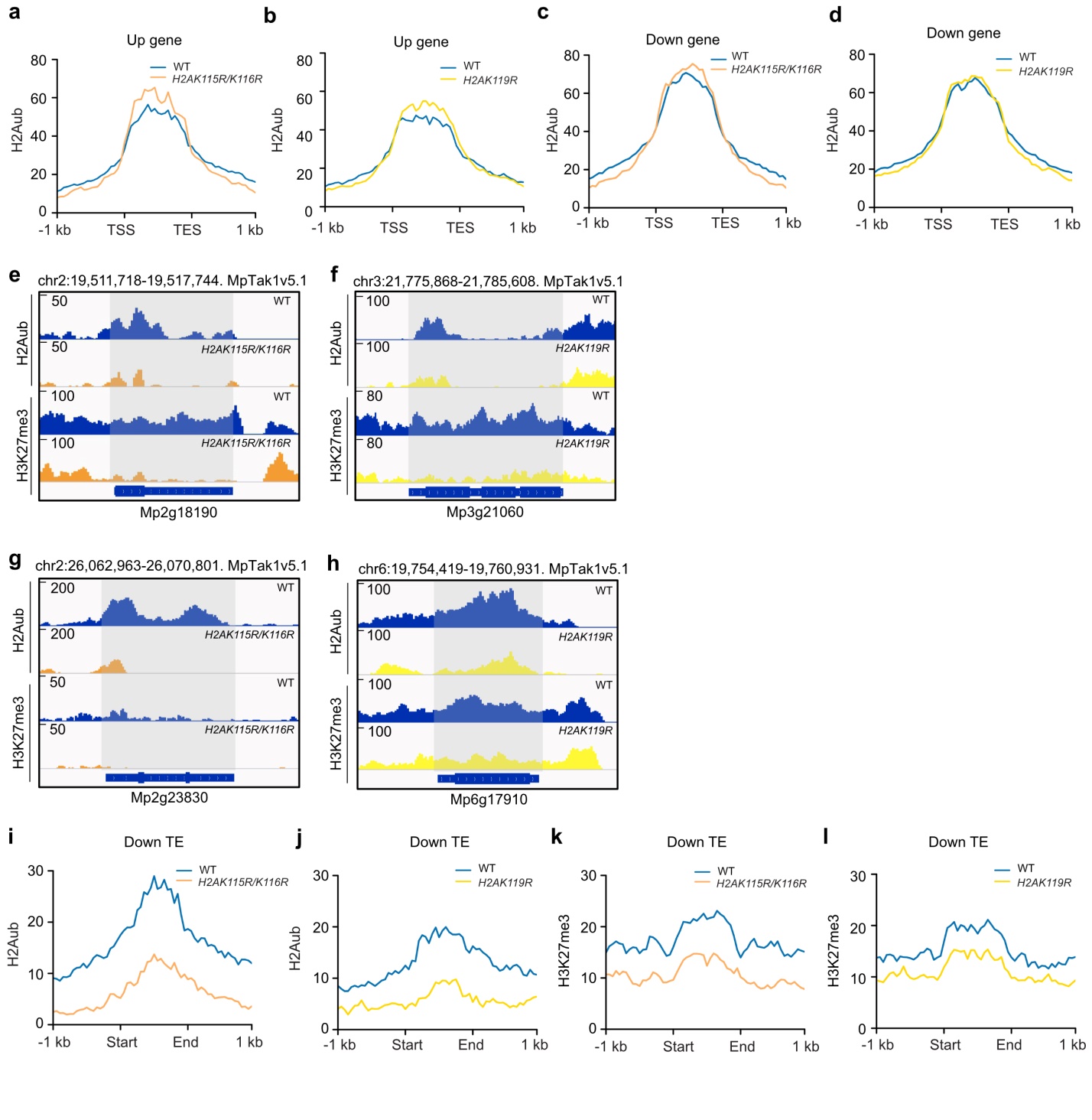


**Figure S6 H2Aub is required for gene repression and activation.**

**a, b.** Metagene plots showing median values of H2Aub levels (RPKM, reads per kilobase per million mapped reads) on upregulated genes in *Mph2a;H2AK115R/K116R* (*H2AK115R/K116R)* mutants (a) and *Mph2a;H2AK119R* (*H2AK119R*) mutants (b). **c, d.** Metagene plots showing median values of H2Aub levels (RPKM) on downregulated genes in *Mph2a;H2AK115R/K116R* mutants (c) and *Mph2a;H2AK119R* mutants (d). **e, f.** Genomic snapshots of H2Aub and H3K27me3 levels on upregulated genes in *Mph2a;H2AK115R/K116R* (e) and *Mph2a;H2AK119R* (f) mutants. **g, h.** Genomic snapshots of H2Aub and H3K27me3 levels on downregulated genes in *Mph2a;H2AK115R/K116R* (g) and *Mph2a;H2AK119R* (h) mutants. **i, j.** Metaplots showing median values of H2Aub levels (RPKM) of downregulated TEs in *Mph2a;H2AK115R/K116R* (i) and *Mph2a;H2AK119R* (j) mutants. **k, l.** Metaplots showing the median values of H3K27me3 levels (RPKM) on downregulated TEs in *Mph2a;H2AK115R/K116R* (k) and *Mph2a;H2AK119R* (l) mutants.


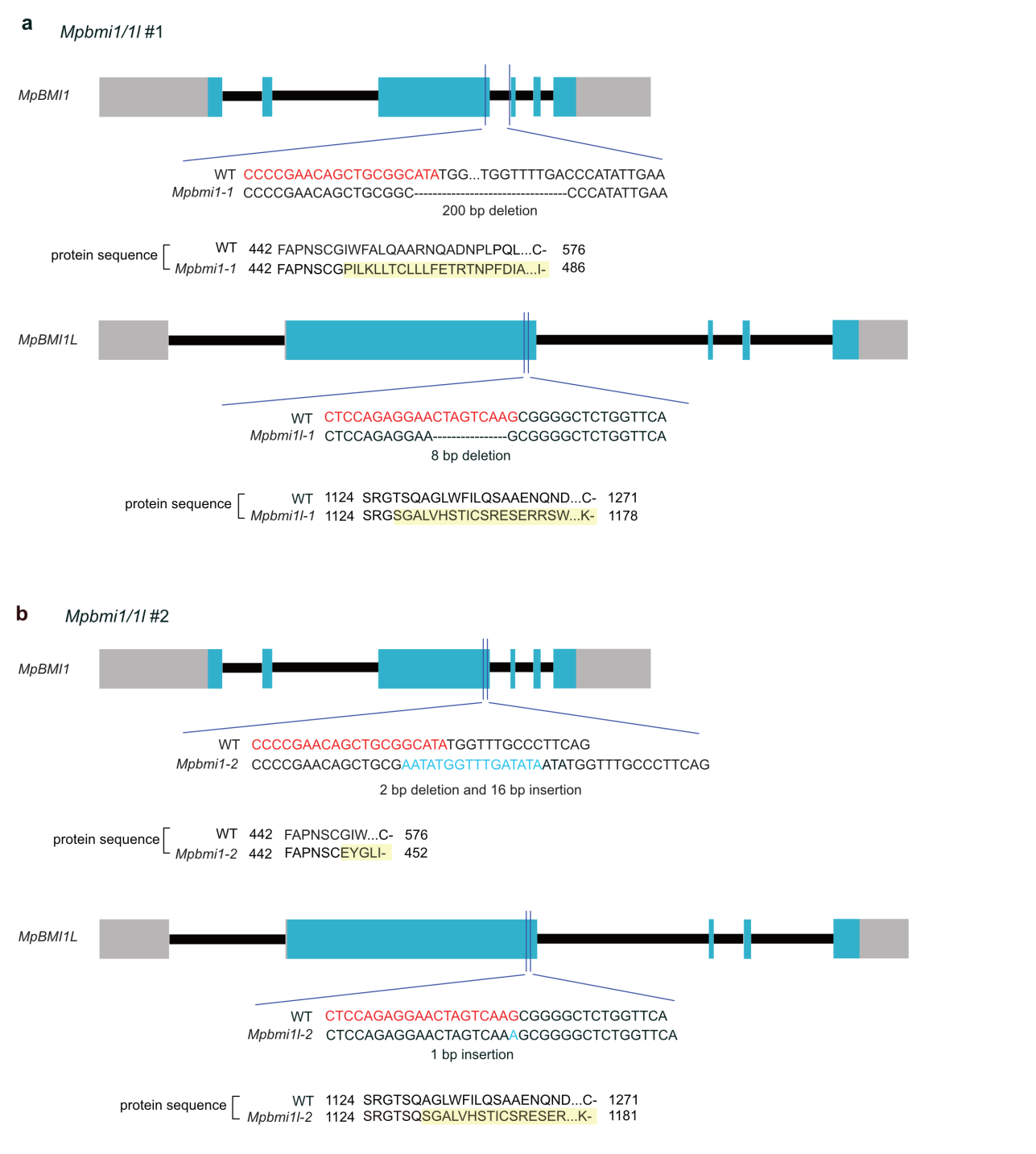


**Figure S7 Characterization of strong double mutant allele combinations in *MpBMI1/1L.***

**a, b.** Nucleotide and protein sequence alignments of wild-type (WT) and mutant alleles of MpBMI1 and MpBMI1L. *Mpbmi1/1l* #1 (a) contains mutant alleles *Mpbmi1-1* and *Mpbmi1l-1. Mpbmi1/1l* #2 (b) contains mutant alleles *Mpbmi1-2* and *Mpbmi1l-2.* gRNA sequences are shown in red. Newly inserted nucleotides are shown in light blue. Nonsense protein sequences are shaded in yellow. “-” refers to the stop codon.


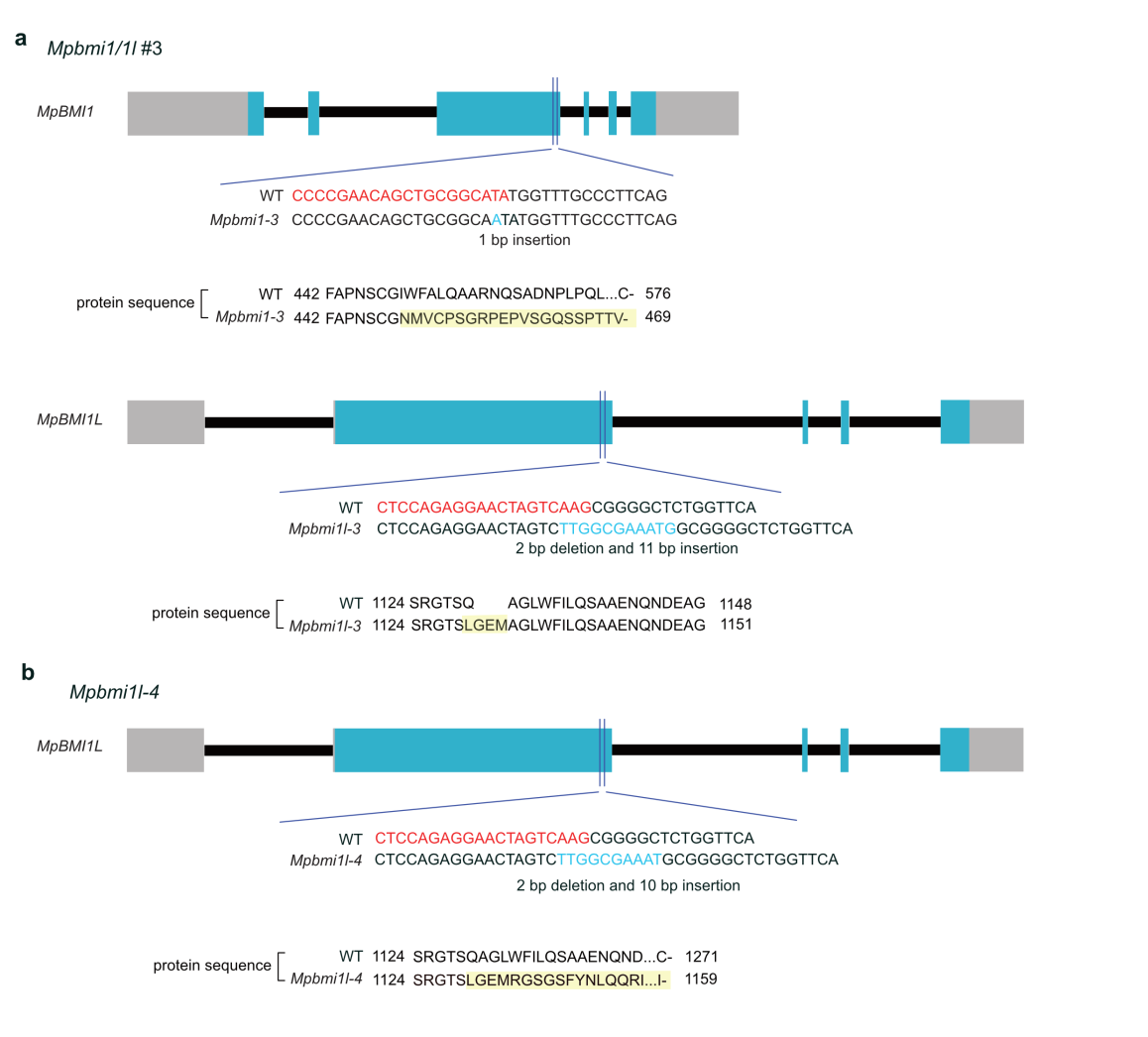


**Figure S8 Characterization of weak double mutant allele combinations in *MpBMI1/1L.***

**a, b.** Nucleotide and protein sequence alignments of wild-type (WT) and mutant alleles of MpBMI1 and MpBMI1L. *Mpbmi1/1l* #3 (a) contains mutant alleles *Mpbmi1-3* and *Mpbmi1l-3*. *Mpbmi1l-4* (b) is a single mutant. gRNA sequences are shown in red. Newly inserted nucleotides are shown in light blue. Nonsense protein sequences are shaded in yellow. “-” refers to the stop codon.


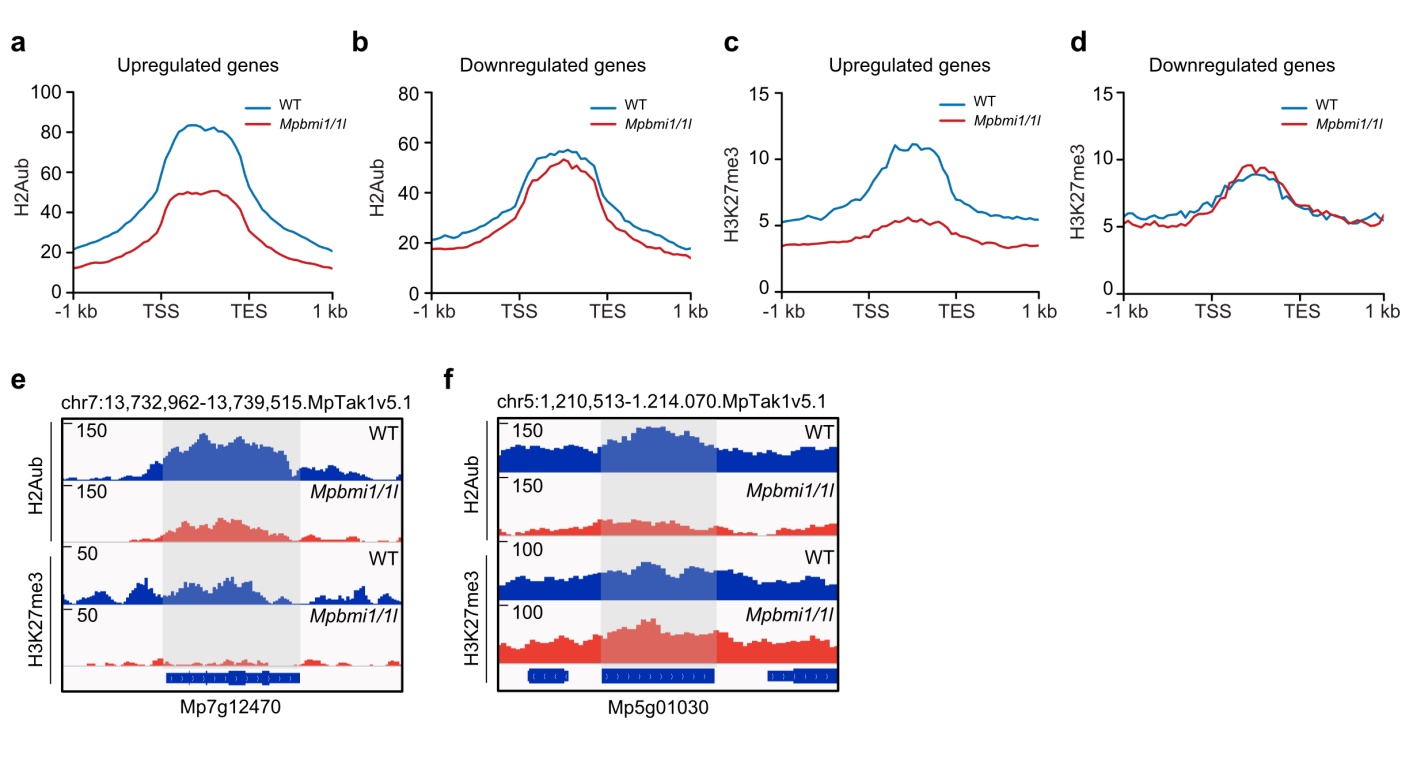


**Figure S9 Deposition of H2Aub is affected on upregulated and downregulated genes by loss of MpBMI1/1L.**

**a, b.** Metagene plots showing median values of H2Aub levels (RPKM, reads per kilobase per million mapped reads) on upregulated (a) and downregulated (b) genes in WT and *Mpbmi1/1l* mutants. **c, d.** Metagene plots showing median values of H3K27me3 levels (RPKM) on upregulated (c) and downregulated (d) genes in WT and *Mpbmi1/1l* mutants. **e, f.** Genomic snapshots of H2Aub and H3K27me3 levels on an upregulated (e) and a downregulated gene (f) in *Mpbmi1/1l* mutants in WT and *Mpbmi1/1l* mutants.


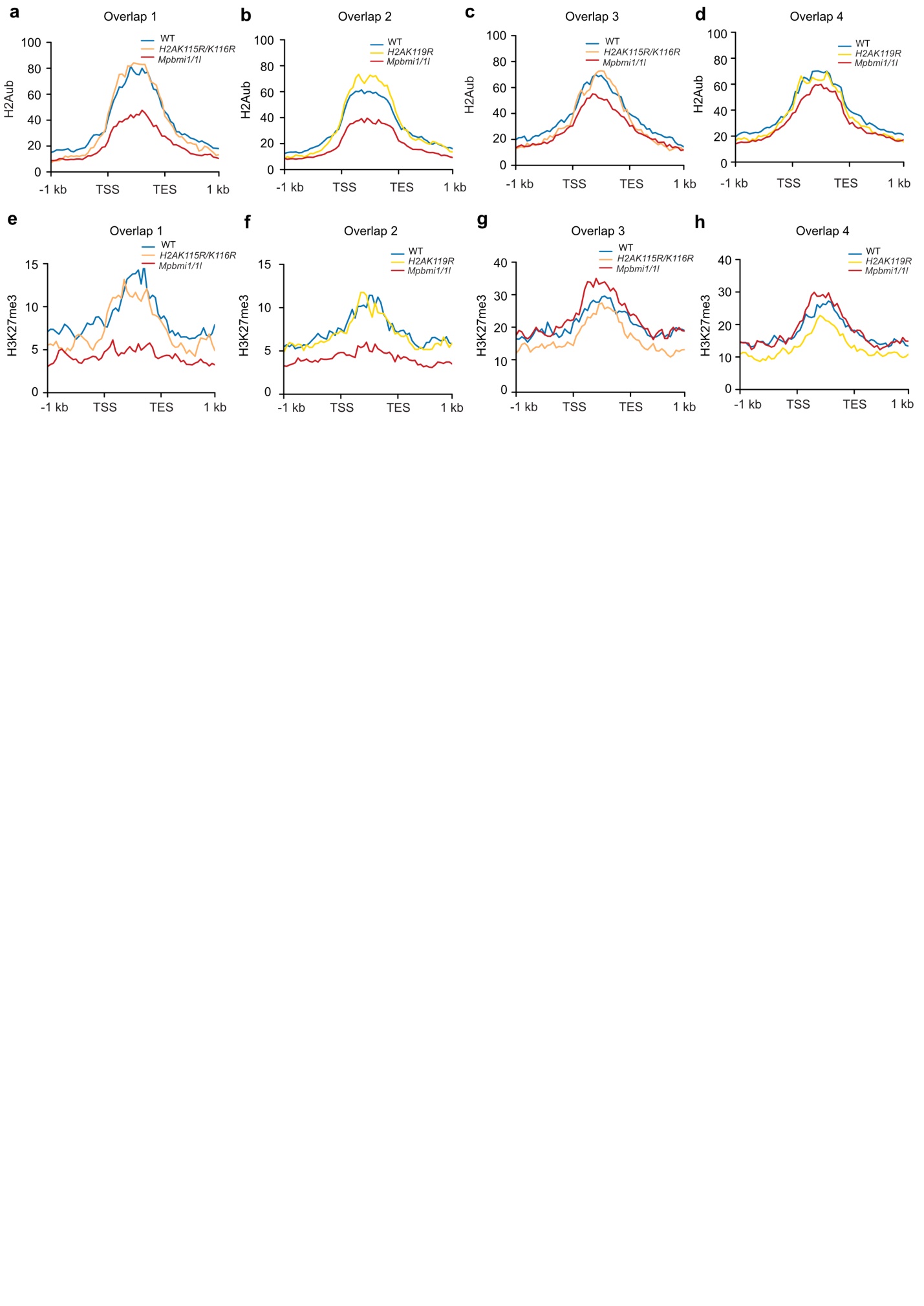


**Figure S10 PRC1-mediated gene repression and activation depends on H2Aub.**

**a-d,** Metagene plots showing median values of H2Aub levels (RPKM, reads per kilobase per million mapped reads) on genes in the group of overlap 1 (a), overlap 2 (b), overlap 3 (c) and overlap 4 (d) in WT, *Mph2a;H2AK115R/K116R* (*H2AK115R/K116R*), *Mph2a;H2AK119R* (*H2AK119R*) and *Mpbmi1/1l* mutants. **e-h.** Metagene plots showing the median values of H3K27me3 levels (RPKM) on genes in the group of overlap 1 (e), overlap 2 (f), overlap 3 (g) and overlap 4 (h) in WT, *Mph2a;H2AK115R/K116R* (*H2AK115R/K116R*), *Mph2a;H2AK119R* (*H2AK119R*) and *Mpbmi1/1l* mutants.

**
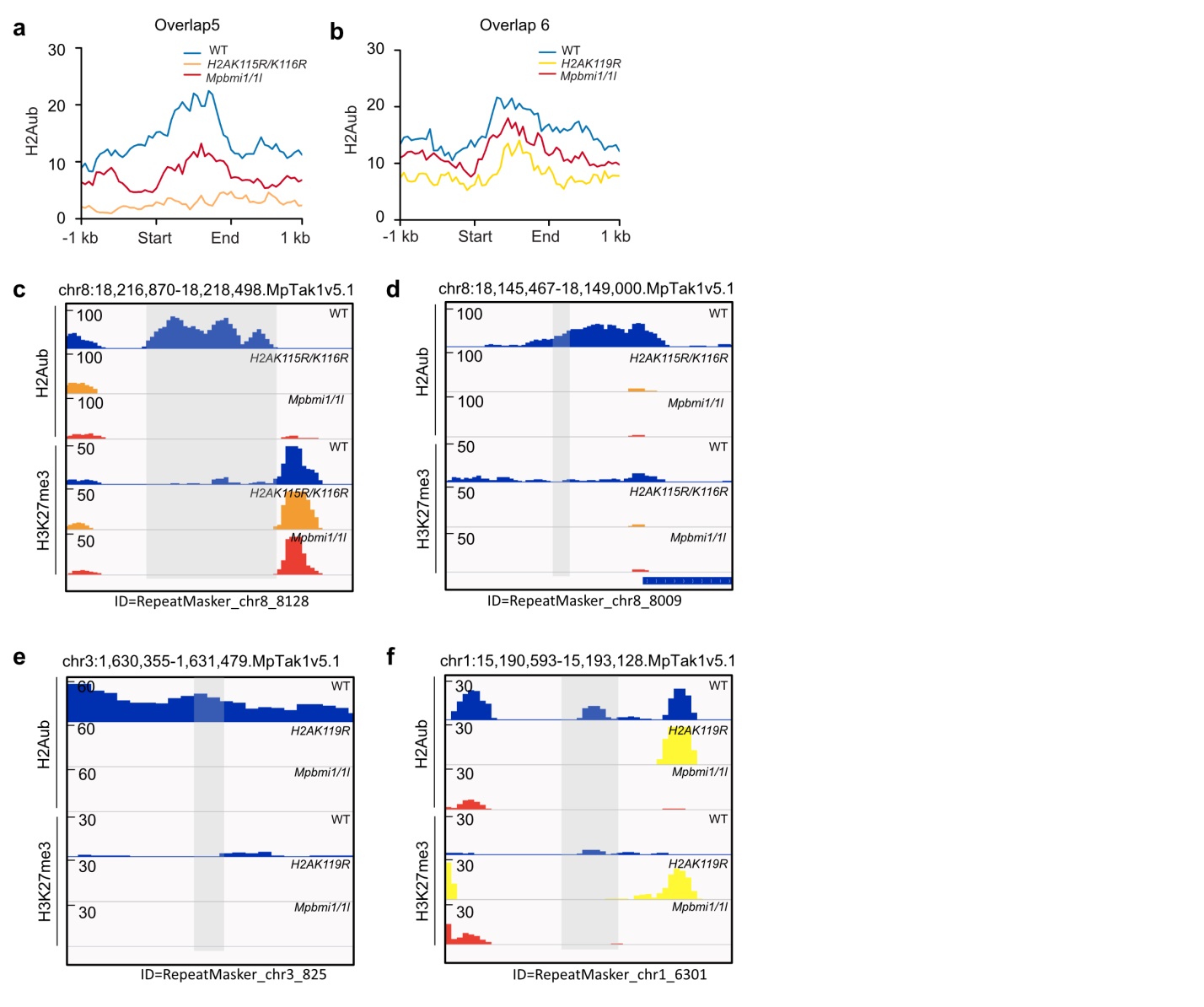
**

**Figure S11 H2Aub contributes to PRC1-mediated transposable element activation.**

**a, b.** Metaplots showing median values of H2Aub levels (RPKM, reads per kilobase per million mapped reads) of commonly downregulated TEs in the group of overlap 5 (a) and overlap 6 (b) in WT, *Mph2a;H2AK115R/K116R* (*H2AK115R/K116R*), *Mph2a;H2AK119R* (*H2AK119R*) and *Mpbmi1/1l* mutants. **c, d.** Genomic snapshots of H2Aub and H3K27me3 levels (RPKM) on two downregulated TEs in *Mph2a;H2AK115R/K116R* mutants in WT, *Mph2a;H2AK115R/K116R* (*H2AK115R/K116R*), and *Mpbmi1/1l* mutants. **e, f.** Genomic snapshots of H2Aub and H3K27me3 levels on two downregulated TEs in *Mph2a;H2AK119R* mutants in WT, *Mph2a;H2AK119R* (*H2AK119R*) and *Mpbmi1/1l* mutants.


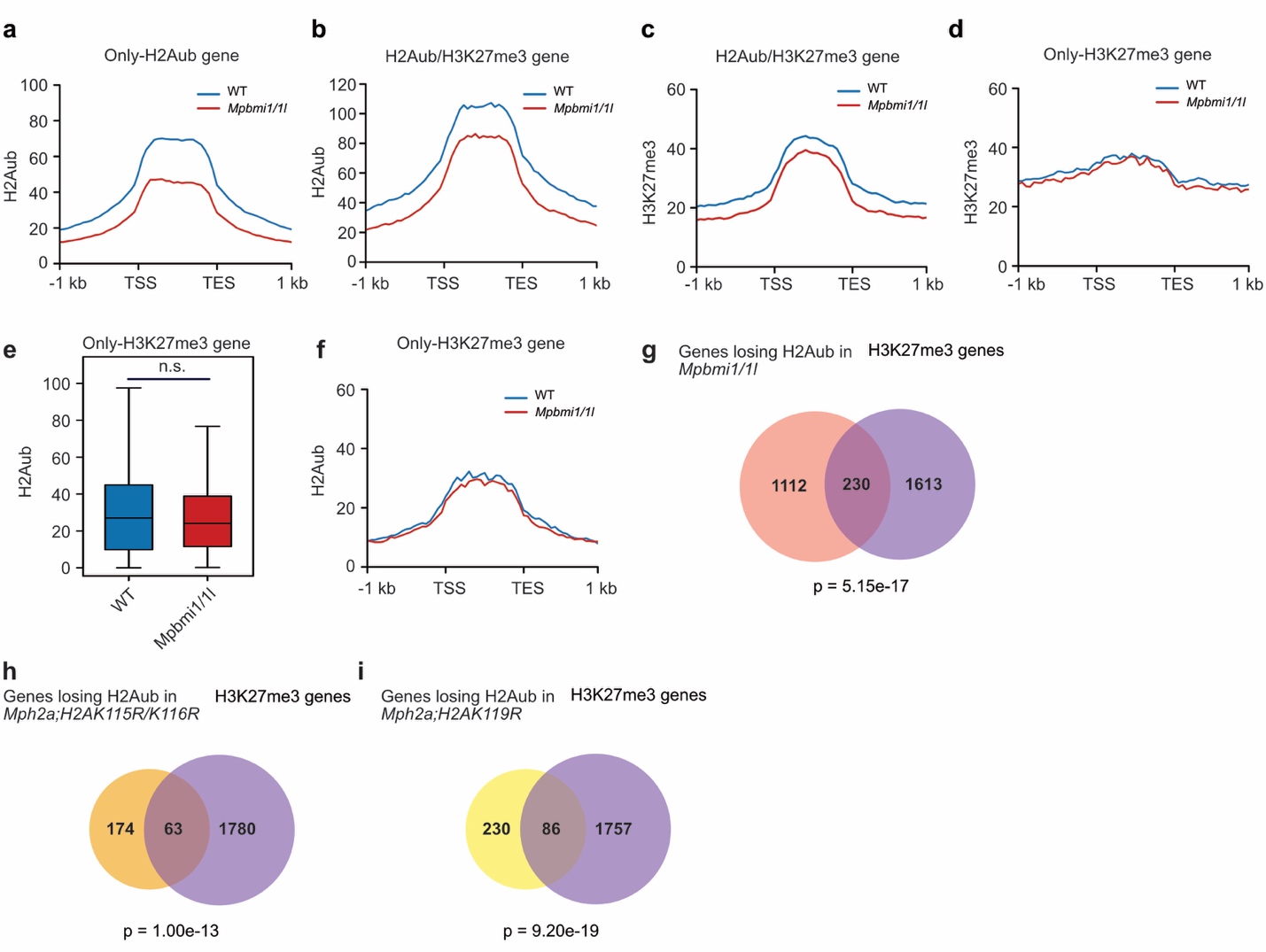


**Figure S12 H2Aub and H3K27me3 deposition are affected by loss of MpBMI1/1L.**

**a, b.** Metagene plots showing median values of H2Aub levels (RPKM, reads per kilobase per million mapped reads) on only-H2Aub marked genes (a) and H2Aub/H3K27me3 genes (b) in wild type (WT) and *Mpbmi1/1l* mutants. **c, d.** Metagene plots showing median values of H3K27me3 levels (RPKM) on H2Aub/H3K27me3 marked genes (c) and only-H3K27me3 marked genes (d) in WT and *Mpbmi1/1l* mutants. **e.** Boxplot showing H2Aub levels on only-H3K27me3 genes. H2Aub levels were calculated as the average RPKM from 1 kb upstream of the transcriptional start to the transcriptional end of genes. Boxes show medians and the interquartile range, and error bars show the full range excluding outliers. n.s., not significant (Wilcoxon test). **f.** Metagene plots showing median values of H2Aub levels on only-H3K27me3 genes. **g.** Venn diagram showing overlap of genes losing H2Aub in *Mpbmi1/1l* mutant and H3K27me3 marked genes. **h.** Venn diagram showing overlap of genes losing H2Aub in *Mph2a;H2AK115R/K116R* mutant and H3K27me3 marked genes. **i.** Venn diagram showing overlap of genes losing H2Aub in *Mph2a;H2AK119R* mutant and H3K27me3 marked genes. Significance was tested using a Hypergeometric test.


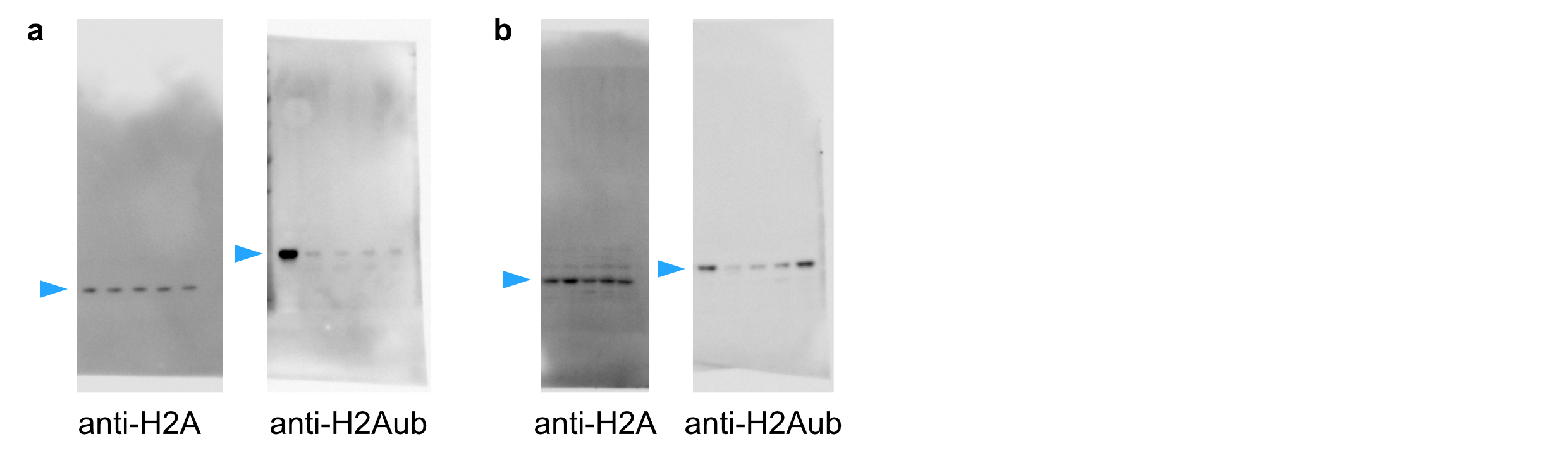


**Figure S13 Western blots of H2A and H2Aub**

**a.** Uncropped image of Fig. 1b. Western blot of bulk H2A and H2Aub levels in wild type (WT), *Mph2a;H2AK115R/K116R* #1, *Mph2a;H2AK115R/K116R* #2, *Mph2a;H2AK119R* #1 and *Mph2a;H2AK119R* #2 mutants. **b.** Uncropped image of Fig. 3i. Western blot showing the bulk H2A and H2Aub levels in WT, *Mpbmi1/1l* #1, *Mpbmi1/1l* #2, *Mpbmi1/1l* #3 and *Mpbmi1l-4* mutants.


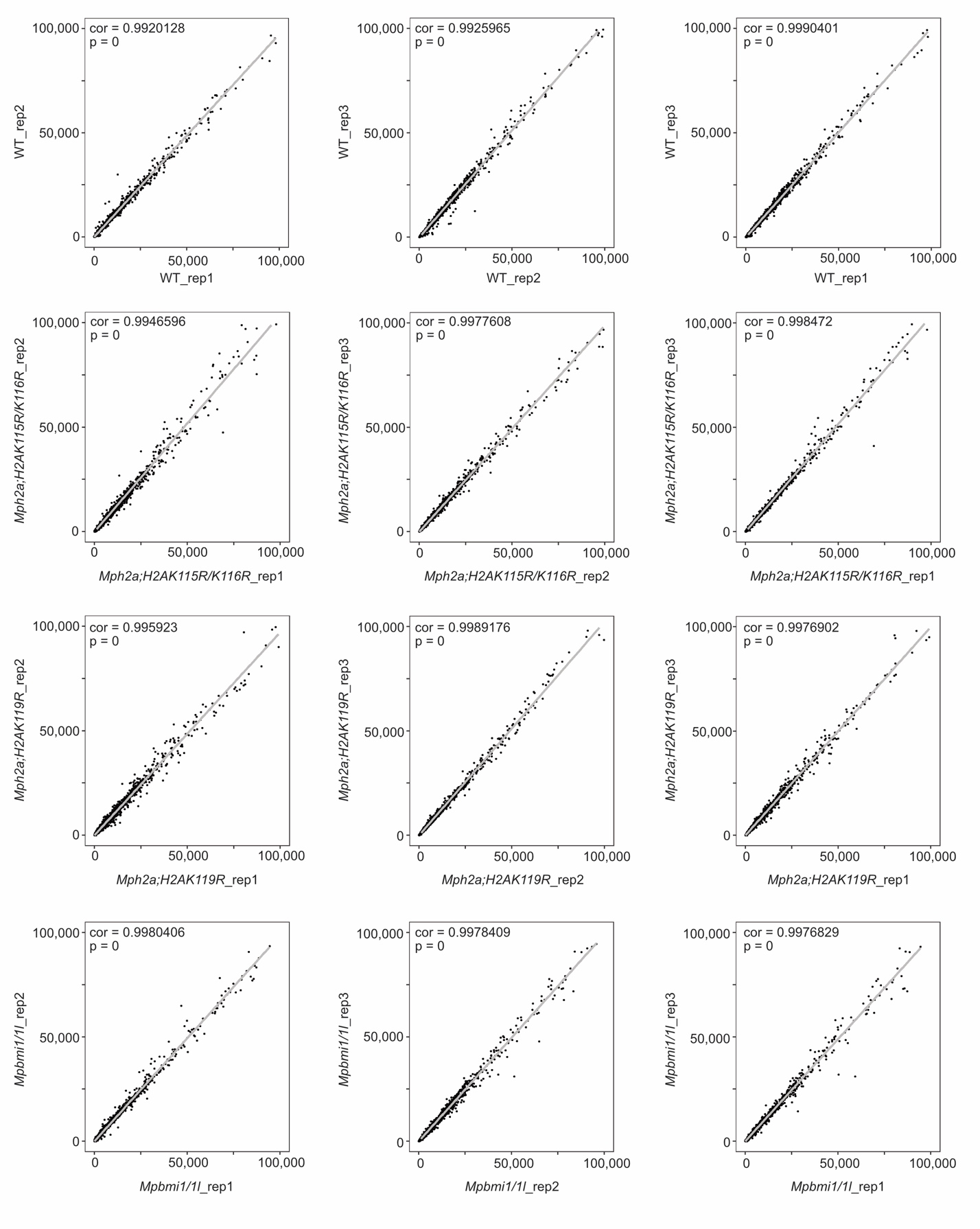


**Figure S14 Scatter plots comparing RNA-seq triplicates.**

Scatter plots of RPKM (reads per kilobase per million mapped reads) from indicated samples. cor, Pearson correlation coefficient.


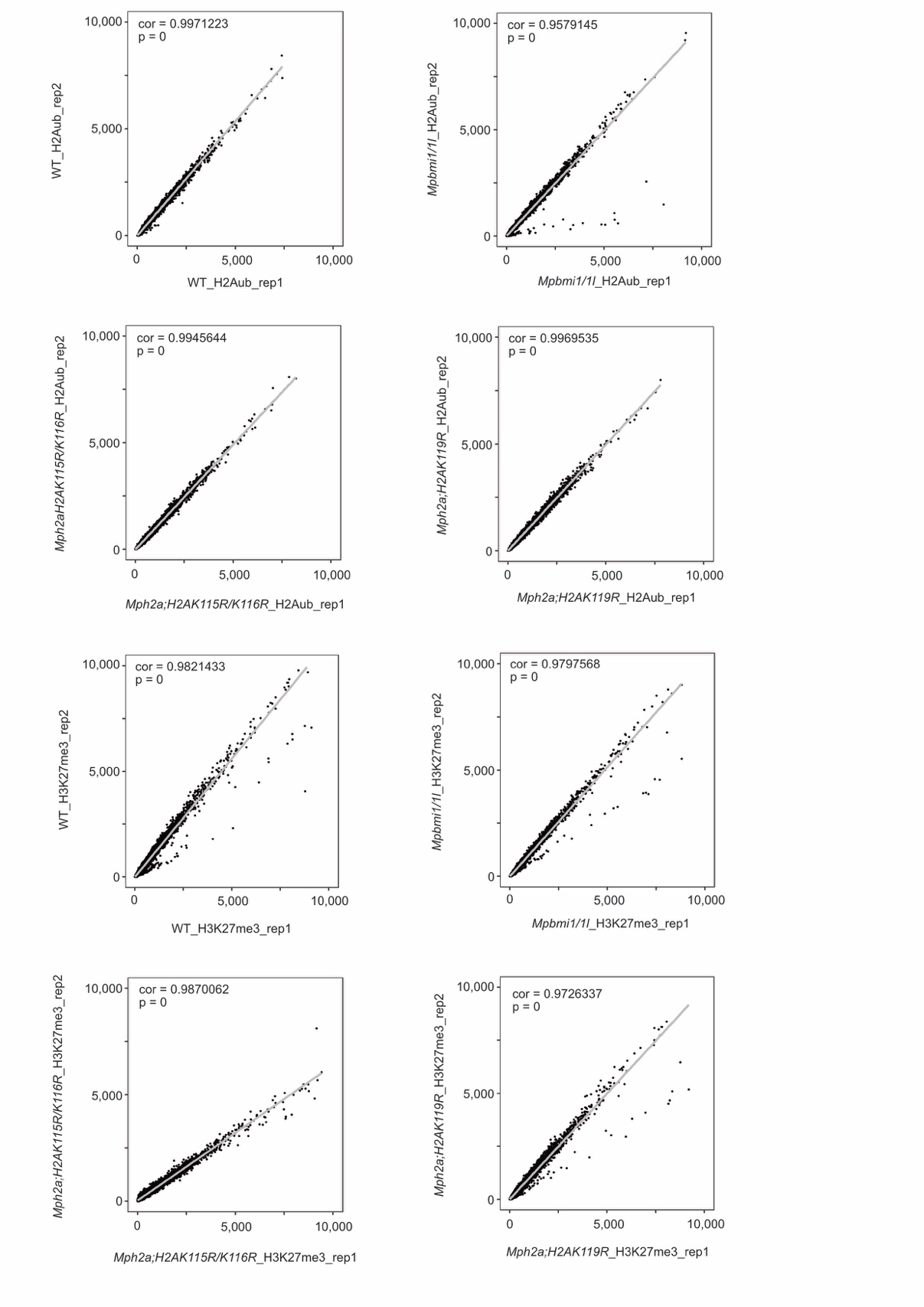


**Figure S15 Scatter plots comparing ChIP-seq replicates.**

Scatter plots of RPKM (reads per kilobase per million mapped reads) in detected peaks from indicated samples. cor, Pearson correlation coefficient.
