# Supplementary material for: H2A ubiquitination is essential for Polycomb Repressive Complex 1-mediated gene regulation in Marchantia polymorpha
Source: Genome Biol. 2021 Sep 1;22:253. doi: 10.1186/s13059-021-02476-y (PMC8408974; doi:10.1186/s13059-021-02476-y)
Supplement: Supplementary file 12 — Additional file 12. Review history. [file 13059_2021_2476_MOESM12_ESM.docx]

Review History

**First round of review**

**Reviewer 1**

**Are you able to assess all statistics in the manuscript, including the appropriateness of statistical tests used?**

Yes, and I have assessed the statistics in my report.

**Comments to author:**

In this manuscript, the authors examined the function of H2Aub in Marchantia by profiling H2Aub, H3K27me3 and transcriptome using point mutated canonical H2A and bmi1 mutants. The results show that H2Aub is required for H3K27me3 deposition and in addition suggest the association of H2Aub with both gene repression and activation. The use of H2A mutants with mutations at potential ubiquitination sites to dissect the direct function of H2Aub is sort of interesting, but most of the findings have been revealed previously in Arabidopsis as the authors also suggested, and this study rather confirmed the conserved role of H2Aub in early land plants. Hence, some new insights into the function or regulatory mechanisms of H2Aub and polycomb silencing would be expected for journals like Genome Biology.

Major concerns

1. This study used an anti-H2Aub antibody (8280S, CST) originally raised against monoubiquitinated human H2A peptide. The sequences of Marchantia H2A and its potential ubiquitination sites substantially differ from that of Arabidopsis and human H2A. Hence, it would be necessary to determine the efficiency and specificity of this antibody in Marchantia. For instance, whether this antibody could effectively recognize Marchantia H2Aub without any cross activity with H2Bub or other chromatin-bound proteins. Some tests with synthesized peptides may help to address this issue.

2. In several figures, the authors calculated RPKM values of H2Aub and H3K27me3 at genes (or gene upstream?). It is stated in the figure captions that "RPKM from 1 kb upstream of the transcriptional start to the transcriptional start of genes" were calculated; in this case 1kb promoter regions were used for calculation. Similarly, in line 132, it states that H2Aub at only promoter regions were used for analysis. However, in the methods part, it states "H2Aub and H3K27me3 scores (RPKM) of genes in boxplots were calculated as the average RPKM from 2 kb upstream of the transcriptional start to the transcriptional end of genes". Hence it is contradictory and confusing. In addition, as H3K27me3 is predominantly localized at the gene body in Arabidopsis, and in Marchantia a large portion of H3K27me3 and H2Aub localizes at gene body (Fig S3), the authors should explain why only the gene upstream or gene upstream plus gene body is used for the analysis. Also it would be better for the authors to show the metagene plots together with the box plots for a more comprehensive interpretation of the data.

3. The use of "all marked genes" (gene with either H2Aub or H3K27me3) for the calculation of H2Aub or H3K27me3 in several figures seems is not appropriate. For instance, in Fig1l, calculation of the H3K27me3 levels with the majority of the loci carry no H3K27me3 (n=3705) may not be suitable. Instead, the comparison of H3K27me3 changes at only-H3K27me3 marked genes (n=636) and H3K27me3/H2Aub genes (n=1260) could be better to reveal the dependence of H3K27me3 on H2Aub.

Minor concerns

1. Fig 2c and 3i show the percentages of only-H2Aub and H2Aub/H3K27me3 marked genes in expression mis-regulated genes. How about only-H3K27me3 marked genes?

2. Fig2j shows that H2Aub marked TEs are more expressed than only-H3K27me3 marked TEs. How about their impacts on the expression levels of protein-coding genes?

3. There are several typos and errors in the text and figures. For instance, line 63 "ubiquitination of", line 221 "whether this requirement if evolutionary conserved", Fig 2d-g "H2A (RPKM)", Fig 5e "Overlap 3/4". The authors are suggested to thoroughly examine the manuscript.

**Reviewer 2**

**Are you able to assess all statistics in the manuscript, including the appropriateness of statistical tests used?**

No, I do not feel adequately qualified to assess the statistics.

**Comments to author:**

Main results

The manuscript reports the analysis of PRC1 activity in histone H2A monoubiquitination in Marchantia polymorpha. The question is mainly addressed by the mean of point mutation of the single histone H2A isoform, thereby prevent its monoubiquitination by PRC1, or by mutating a MpBMI1/L PRC1 component subunit. The authors notably perform RNA-seq analyses in these different genetic contexts to assess some functional interplays between H2Aub and gene/TE expression. In a second part, the authors analyse H3K27me3 profiles to identify the influence of H2Aub or PRC1 activity on the possible links between the PRC1 and PRC2 complexes.

Detailed review report to the editor and authors.

The first results showed by the authors is the influence of point mutation of H2A on Marchantia growth and on H2Aub and H3K27me3 epigenomic profile in Marchantia. When comparing the results on two different mutated lines (Mph2a;H2AK115R/K116R and Mph2a;H2AK119R), despite observing in both cases a decrease of H2Aub compared to WT (Figure 1i and Figure 1j) we miss some information to assess the quality of the ChIP-seq profiles and absence of artifactual background that might bias these analyses. Among these controls, we miss the number of genes affected to appreciate the extent of the mutation effect. We would also benefit of the Venn in fig 4a and 4e showing the overlap of genes analysed in figure 1i and 1j. The number of TEs and intergenic regions that were called in te peak analysis (in figure S2 over 4000 peaks are called in this study, and only 1896 overlap with genes fig 1k) and showing some IGV screenshots of representative regions undergoing H3K27me3 reduction in the h2a_ub lines would help appreciating the quality of the ChIP-seq profiles and of the difference observed.

When investigating the influence of H2Aub on transcription activation the authors describe a decreased level in the promoter regions of DEGs being down or upregulated in the mutant lines (Fig 2d-2g) the authors indicate that this support a role of H2Aub in both repression and activation (line 133), it would be interesting to further present this hypothesis in the discussion and possibly link to an effect of PRC1 on H2A.Z as in Arabidopsis. Also, to corroborate this observation it would be interesting to show the overlap of up and down genes in fig 2a and fig2b to appreciate the different influence of the 2 lines. While illustrating the changes of H2Aub over different genes categories in the two lines (Fig 2d,e,f,g,) we miss the extent of changes of H2Aub, as well as H3K27me3, over different classes of TEs identified in figures 2h and 2i. These analyses would help corroborating the link between TE repression and H2Aub proposed in line 138.

Last, in these comparisons is-there a difference of H2Aub or H3K27me3 profile in the up and down DEGs, which would explain a differential influence of PRC1 on their expression?

While a second approach of mutating the Mpbmi1/1l is very interesting to untangle the different influences of PRC1 occupancy and H2Aub marking, it would be interesting to better quantify the role of these two factors on the general level of H2Aub, possibly by quantifying the WB (Fig3i) in different replicates and testing other histone marks, in particular H3K27me3 but eventually also H3K4me" or H3K36me3.

In figure 4, generating directly an overlap by comparing the 3 groups of genes, instead of 2 by 2, could better drive to the results. Despite showing the PRKM level (Figures 4i,j,k,l) of the different overlapping genes, we miss the information on H2Aub and H3K27me3 profiles these shared genes.

When investigating the role of H2Aub and more generally PRC1 complex on TE silencing, the authors should clarify if the intergenic regions indicated at line 194 are actually overlapping the 900 upregulated TEs indicated in line 195, and also how they extracted the list of TEs upregulated in the h2a_ub mutants (line 196 and Fig5c-5e) if by intersection of the two lists. In figure 5c to 5g the authors focus on TEs upregulated, despite showing a similar or even higher number of TEs being downregulated in the h2a_ub mutants (Fig2h and 2i), we thus wondered if a similar effect would be appreciated over downregulated TEs and how much these tendencies are supported by significant transcript levels. Given the illustrated link with H2Aub it would interesting to plot using metagenes, boxplots or showing IGV profiles of representative TEs, the changes in the profile both in H2Aub and H3K27me3.

In brief, while the authors illustrated an interplay between H2Aub and PRC2 mediated H3K27me3 over genic and intergenic regions, this would be better substantiated by further graphic representation of the results. As mentioned above, i figure 6a to 6d the authors should again use a similar scale to better appreciate the differences between the different classes, as well as stating clearly the number of genes/regions underlying the different plots. As for figure 1l the authors could further substantiate these conclusions by plotting the level of H3K27me3 over all peaks, or all genes, or all intergenic regions to underling if the influence of PRC1 mutation is limited over the H2Aub/H3K27me3 overlapping regions or rather a general genome wide influence. Heatmap profile of the different regions would also help appreciating if the changes of marking are specifically located over gene promoters or rather span over the all annotation of genes/ intergenic regions. The IGV profiles are quite zoomed in to being able to appreciate the quality of the Chip-seq data, further regions and zoom out profile would help illustrating how the peaks are separated from the background noise. Also adding a track with genes, or IGV screenshot of representative genes, would help appreciating the differences in marking between genic regions and intergenic regions and further help to corroborate the conclusions.

Indeed, in the discussion the authors drive some strong conclusion, such as that PRC1-catalysed H2Aub is required for TE repression in Marchantia (line 237) without exploring the role over TE expression and without showing a specific link of changes of H2Aub or H3K27me3 level over the TE upregulated. These examples illustrate the need of better substantiate and more clearly link the conclusions with the presentation of the results.

Minor points:

- How look like the Marchantia H2A protein, as compared to the different H2A variants found in Arabidopsis (H2A.Z, H2A.W, H2A.X). In particular, it would be interesting to test H2A.Z ubiquitination in the Mpbmi1/1l mutant line to gain a better understanding of its phenotype.

- When citing literature, please better highlight when describing situations in Marchantia or in other species such as Arabidopsis. This can be confusing at different places along the manuscript.

- In Figure1 there are large differences of scales in the box-plots of gene categories "all marked genes" and "H2Aub/H3K27me3 marked genes" as compared with the "intergenic regions". Using the same scale for all boxplots would better illustrate the amplitude of the different changes.

- Minor mistake, figure 5e seems to represent a Venn comparison of overlap between 5 and 6 (and not 3 and 4).

- Police size in the figures is well too small.

**Authors Response**

**Point-by-point responses to the reviewers’ comments:**

**Reviewer #1:**

In this manuscript, the authors examined the function of H2Aub in Marchantia by profiling H2Aub, H3K27me3 and transcriptome using point mutated canonical H2A and bmi1 mutants. The results show that H2Aub is required for H3K27me3 deposition and in addition suggest the association of H2Aub with both gene repression and activation. The use of H2A mutants with mutations at potential ubiquitination sites to dissect the direct function of H2Aub is sort of interesting, but most of the findings have been revealed previously in Arabidopsis as the authors also suggested, and this study rather confirmed the conserved role of H2Aub in early land plants. Hence, some new insights into the function or regulatory mechanisms of H2Aub and polycomb silencing would be expected for journals like Genome Biology.

*Reply: Our manuscript reports three novel findings that go beyond mere confirmatory knowledge:*

*(i) Previous reports revealed that PRC2 binding to chromatin requires PRC1 but not H2Aub in Drosophila (Kahn et al., 2016). In Arabidopsis, PRC1 was shown to interact with PRC2, but the direct contribution of H2Aub for H3K27me3 deposition remains to be established, since it is possible that PRC1 regulates H3K27me3 independently of H2Aub. By analyzing h2a_ub mutants, we were able to demonstrate the direct contribution of H2Aub for H3K27me3 targeting, which is novel and worth reporting.*

*(ii) H2Aub is not required for repression of PRC1 target genes in Drosophila (Pengelly et al., 2015). In contrast, reports in mouse embryonic stem cells showed that H2Aub is essential in Polycomb-mediated repression (Tamburri et al., 2020); therefore, the role of H2Aub is still ambiguous. H2Aub is associated with permissively accessible chromatin in Arabidopsis (Yin et al., 2021); however, the connection between H2Aub with transcriptional activation remains unknown. Furthermore, PRC1-mediated silencing was reported on some target genes in Arabidopsis; nevertheless, whether PRC1 catalytic activity is generally required for Polycomb-mediated gene silencing in plants still needs to be established. We found PRC1-mediated H2Aub contributes directly to gene silencing and activation and showed that H2Aub-mediated gene activation can be direct and independent of H3K27me3.*

*(iii) H3K27me3 is enriched in intergenic region in Marchantia (Bowman et al., 2017), differing from the mainly genic distribution of H3K27me3 in Arabidopsis. However, whether PRC1 and H2Aub contribute to transposable element (TE) transcriptional regulation remains to be investigated. We found PRC1-mediated H2Aub is required for TE activation independently of H3K27me3, uncovering a new role for PRC1-mediated H2Aub, which is distinct from the Polycomb regulatory network.*

Major concerns

1. This study used an anti-H2Aub antibody (8280S, CST) originally raised against monoubiquitinated human H2A peptide. The sequences of Marchantia H2A and its potential ubiquitination sites substantially differ from that of Arabidopsis and human H2A. Hence, it would be necessary to determine the efficiency and specificity of this antibody in Marchantia. For instance, whether this antibody could effectively recognize Marchantia H2Aub without any cross activity with H2Bub or other chromatin-bound proteins. Some tests with synthesized peptides may help to address this issue.

*Reply: The C-terminal regions of H2A proteins of human (Hs), Arabidopsis (At) and Marchantia (Mp) are conserved (see the figure above), including the ubiquitinated lysine residues (purple arrowheads). In contrast, the C-terminal sequence of MpH2B is largely distinct from H2A, which is therefore highly unlikely to be detected by the anti-H2Aub antibody. Moreover, the H2Aub level was significantly decreased in h2a_ub and Mpbmi1/1l mutants (Fig. 1b and 3i), revealing that anti-H2Aub specifically recognized ubiquitinated H2A. In Arabidopsis and rice, HUB1 and HUB2 function as E3 ligases for H2B monoubiquitination (Cao et al., 2015), suggesting that H2Bub is not regulated by MpBMI1/1L. Whether the anti-H2Aub antibody can recognize H2A.Zub is unknown, but we only observed one band on western blots (Fig. 1b and 3i), likely corresponding to H2Aub. Furthermore, H2A.Zub will not be affected in the h2a_ub mutants, which have substituted lysine residues in H2A. In summary, this data strongly suggests that the anti-H2Aub antibody does specifically recognize H2Aub in Marchantia.*

2. In several figures, the authors calculated RPKM values of H2Aub and H3K27me3 at genes (or gene upstream?). It is stated in the figure captions that "RPKM from 1 kb upstream of the transcriptional start to the transcriptional start of genes" were calculated; in this case 1kb promoter regions were used for calculation. Similarly, in line 132, it states that H2Aub at only promoter regions were used for analysis. However, in the methods part, it states "H2Aub and H3K27me3 scores (RPKM) of genes in boxplots were calculated as the average RPKM from 2 kb upstream of the transcriptional start to the transcriptional end of genes". Hence it is contradictory and confusing. In addition, as H3K27me3 is predominantly localized at the gene body in Arabidopsis, and in Marchantia a large portion of H3K27me3 and H2Aub localizes at gene body (Fig S3), the authors should explain why only the gene upstream or gene upstream plus gene body is used for the analysis. Also it would be better for the authors to show the metagene plots together with the box plots for a more comprehensive interpretation of the data.

*Reply: Thanks for pointing out this inconsistency. In Fig. 1i, 1j, 1k, 1m, 3k, 3i and 6a-6e, to test whether H2Aub directly affects H3K27me3 incorporation in the specified group of genes, we analyzed the region covering 1 kb upstream of the transcriptional start to the transcriptional end of genes. In Fig. 2e-2h and 4g-4n, to test H2Aub and H3K27me3 levels on the deregulated genes in h2a_ub and Mpbmi1/1l mutants, we analyzed the 1 kb promoter region since accessibility of promoter region affects transcription factor binding and transcriptional change. Furthermore, we found that the decrease of H2Aub on deregulated genes in h2a_ub mutants was only detectable in the promoter region, likely due to the redundant ubiquitination of H2AK115/K116 and H2AK119. We have added this information in Line 149-153 (revised version with track changes) and included metagene plots in Fig. S6a-S6d and S10. Reduced H2Aub on upregulated genes in Mpbmi1/1l mutants was observed in both, promoter and gene body region (Fig. S10a-S10b), consistent with the increased transcriptional upregulation in Mpbmi1/1l mutants compared to h2a_ub mutants (Fig.4c and 4d). Metagene plots for the specified groups of genes have been provided in Fig. S3, S6, S9, S10 and S12.*

3. The use of "all marked genes" (gene with either H2Aub or H3K27me3) for the calculation of H2Aub or H3K27me3 in several figures seems is not appropriate. For instance, in Fig1l, calculation of the H3K27me3 levels with the majority of the loci carry no H3K27me3 (n=3705) may not be suitable. Instead, the comparison of H3K27me3 changes at only-H3K27me3 marked genes (n=636) and H3K27me3/H2Aub genes (n=1260) could be better to reveal the dependence of H3K27me3 on H2Aub.

*Reply: We agree with the reviewer and have removed the boxplot showing H3K27me3 level on “all marked genes”. We added a boxplot and metagene plot showing H3K27me3 level on only-H3K27me3 genes in Fig. S3e. We found that H3K27me3 levels decreased, but H2Aub levels increased in only-H3K27me3 genes (Fig. S3f) in h2a_ub mutants. H2Aub was proposed to recruit REF6, (Kralemann et al., 2020), providing an explanation for the observed loss of H3K27me3 despite increased H2Aub. In Fig. 6a, 6d, S12a and S12d, we added boxplots and metagene plots showing H2Aub levels on only-H2Aub genes and H3K27me3 levels on only-H3K27me3 genes in Mpbmi1/1l mutants.*

Minor concerns

1. Fig 2c and 3i show the percentages of only-H2Aub and H2Aub/H3K27me3 marked genes in expression mis-regulated genes. How about only-H3K27me3 marked genes?

*Reply: Only-H3K27me3 marked genes were also enriched among the mis-regulated genes, but were nevertheless only a minor fraction among mis-regulated genes. We added this information to Fig. 2c and 3m. The enrichment of only-H3K27me3 genes among deregulated genes in h2a_ub mutants is possibly a consequence of the increased H2Aub levels on only-H3K27me3 genes (Fig. S3f).*

2. Fig2j shows that H2Aub marked TEs are more expressed than only-H3K27me3 marked TEs. How about their impacts on the expression levels of protein-coding genes?

*Reply: H2Aub marked genes have significantly higher number of transcriptionally active genes than H2Aub/H3K27me3 and only-H3K27me3 genes. We added this information to Fig. 2d.*

3. There are several typos and errors in the text and figures. For instance, line 63 "ubiquitination of", line 221 "whether this requirement if evolutionary conserved", Fig 2d-g "H2A (RPKM)", Fig 5e "Overlap 3/4". The authors are suggested to thoroughly examine the manuscript.

*Reply: Thanks for pointing this out. We corrected the errors mentioned above and thoroughly examined the manuscript.*

**Reviewer #2:**

Main results

The manuscript reports the analysis of PRC1 activity in histone H2A monoubiquitination in Marchantia polymorpha. The question is mainly addressed by the mean of point mutation of the single histone H2A isoform, thereby prevent its monoubiquitination by PRC1, or by mutating a MpBMI1/L PRC1 component subunit. The authors notably perform RNA-seq analyses in these different genetic contexts to assess some functional interplays between H2Aub and gene/TE expression. In a second part, the authors analyse H3K27me3 profiles to identify the influence of H2Aub or PRC1 activity on the possible links between the PRC1 and PRC2 complexes.

Detailed review report to the editor and authors.

The first results showed by the authors is the influence of point mutation of H2A on Marchantia growth and on H2Aub and H3K27me3 epigenomic profile in Marchantia. When comparing the results on two different mutated lines (Mph2a;H2AK115R/K116R and Mph2a;H2AK119R), despite observing in both cases a decrease of H2Aub compared to WT (Figure 1i and Figure 1j) we miss some information to assess the quality of the ChIP-seq profiles and absence of artifactual background that might bias these analyses. Among these controls, we miss the number of genes affected to appreciate the extent of the mutation effect. We would also benefit of the Venn in fig 4a and 4e showing the overlap of genes analysed in figure 1i and 1j. The number of TEs and intergenic regions that were called in te peak analysis (in figure S2 over 4000 peaks are called in this study, and only 1896 overlap with genes fig 1k) and showing some IGV screenshots of representative regions undergoing H3K27me3 reduction in the h2a_ub lines would help appreciating the quality of the ChIP-seq profiles and of the difference observed.

*Reply: (i) We provided the numbers of genes with differential H2Aub and H3K27me3 peaks in h2a_ub and Mpbmi1/1l mutants in Table S8 and Table S9.*

*(ii) Since the ubiquitination on H2AK115/K116 and H2AK119 is reductant, we did not identify many genes that passed the significance threshold for changes of H2Aub and H3K27me3 in h2a_ub mutants and we therefore did not show the data as Venn diagram. However, we analyzed the H2Aub level on commonly upregulated and downregulated genes in h2a_ub and Mpbmi1/1l mutants and provided this information in Fig. 4g-4j and S10a-S10d. The H2Aub levels were decreased in the promoter region of commonly upregulated and downregulated genes in h2a_ub and Mpbmi1/1l mutants, supporting the idea that H2Aub is required for PRC1-mediated gene repression and activation.*

*(iii) We tested the number of transposable elements marked by H2Aub and H3K27me3. We found H3K27me3 peaks are enriched on more than 10,000 TEs and of those a significant number overlapped with H2Aub marked TEs (Fig. 1n).*

*(iv) We added IGV screen shots in Fig. S3c and S3d, which showed randomly selected regions undergoing H3K27me3 reduction in h2a_ub lines. The IGV screen shots showed clear H2Aub and H3K27me3 peaks compared to the background, supporting the quality of ChIP-seq data.*

When investigating the influence of H2Aub on transcription activation the authors describe a decreased level in the promoter regions of DEGs being down or upregulated in the mutant lines (Fig 2d-2g) the authors indicate that this support a role of H2Aub in both repression and activation (line 133), it would be interesting to further present this hypothesis in the discussion and possibly link to an effect of PRC1 on H2A.Z as in Arabidopsis. Also, to corroborate this observation it would be interesting to show the overlap of up and down genes in fig 2a and fig2b to appreciate the different influence of the 2 lines. While illustrating the changes of H2Aub over different genes categories in the two lines (Fig 2d-2g,) we miss the extent of changes of H2Aub, as well as H3K27me3, over different classes of TEs identified in figures 2h and 2i. These analyses would help corroborating the link between TE repression and H2Aub proposed in line 138.

Last, in these comparisons is-there a difference of H2Aub or H3K27me3 profile in the up and down DEGs, which would explain a differential influence of PRC1 on their expression?

*Reply: (i) It was shown that PRC1 can target H2A.Z and that H2A.Z monoubiquitination correlates with transcriptional repression in Arabidopsis (Gómez-Zambrano et al., 2019). It was furthermore proposed that H2Aub cannot completely compensate for H2A.Zub, which could explain that the effect of mutants in PRC1 components were substantially stronger than the effect in h2a_ub. We elaborated on this possibility in the discussion.*

*(ii) We showed the overlap of upregulated and downregulated gene between the two h2a_ub mutants in Fig. 4a and Fig. 4b.*

*(iii) We tested H2Aub levels on upregulated and downregulated TEs in Fig 2i and Fig 2j (previous Fig. 2h and 2i) and found H2Aub being decreased on the downregulated TEs but not on upregulated TEs (Fig. 2m-2p), indicating that PRC1-mediated H2Aub is required for TE activation, but not for TE repression. The decrease of H2Aub was observed over the whole length of the TEs (Fig. S6i, S6j). H3K27me3 was also decreased on the downregulated TEs (Fig. S6k, S6l), suggesting that H2Aub-mediated TE activation is direct and independent of H3K27me3. We modified our conclusions accordingly.*

*(iv) Due to the redundant function of ubiquitination on H2AK115/K1116 and H2AK119, we tested H2Aub and H3K27me3 levels on the upregulated and downregulated genes in Mpbmi1/1l mutants to reveal the differential effect of PRC1 on these two groups of genes. H2Aub was decreased on both upregulated and downregulated genes (Fig. 3k), but H3K27me3 was only decreased on the upregulated genes in Mpbmi1/1l mutants (Fig. 3l), supporting the idea that PRC1-mediated gene repression relies on H3K27me3, but gene activation can be direct and independent of H3K27me3.*

While a second approach of mutating the Mpbmi1/1l is very interesting to untangle the different influences of PRC1 occupancy and H2Aub marking, it would be interesting to better quantify the role of these two factors on the general level of H2Aub, possibly by quantifying the WB (Fig3i) in different replicates and testing other histone marks, in particular H3K27me3 but eventually also H3K4me" or H3K36me3.

*Reply: Since we have generated ChIP-seq data of H2Aub and H3K27me3 in Mpbmi1/1l mutants in 2 replicates which showed high reproducibility, we think to quantify the WB in different replicates is not necessarily required. Since our study was focussed on understanding the functional requirement of PRC1 catalytic activity, we focussed on H2Aub and H3K27me3, two modifications that are either directly or indirectly linked to PRC1 activity, respectively. We think that testing H3K4me and H3K36me3 goes beyond the scope of this manuscript.*

In figure 4, generating directly an overlap by comparing the 3 groups of genes, instead of 2 by 2, could better drive to the results. Despite showing the PRKM level (Figures 4i,j,k,l) of the different overlapping genes, we miss the information on H2Aub and H3K27me3 profiles these shared genes.

When investigating the role of H2Aub and more generally PRC1 complex on TE silencing, the authors should clarify if the intergenic regions indicated at line 194 are actually overlapping the 900 upregulated TEs indicated in line 195, and also how they extracted the list of TEs upregulated in the h2a_ub mutants (line 196 and Fig5c-5e) if by intersection of the two lists. In figure 5c to 5g the authors focus on TEs upregulated, despite showing a similar or even higher number of TEs being downregulated in the h2a_ub mutants (Fig2h and 2i), we thus wondered if a similar effect would be appreciated over downregulated TEs and how much these tendencies are supported by significant transcript levels. Given the illustrated link with H2Aub it would be interesting to plot using metagenes, boxplots or showing IGV profiles of representative TEs, the changes in the profile both in H2Aub and H3K27me3.

*Reply: (i) We included an overlap by comparing the 3 group of genes in Fig. 4a and Fig. 4b.*

*(ii) We added H2Aub and H3K27me3 levels on commonly upregulated and downregulated genes between h2a_ub and Mpbmi1/1l mutants in Fig. 4g-4n and S10.*

*(iii) To better correlate the role of H2Aub for TE expression, we focused on H2Aub and H3K27me3 marked TEs instead of intergenic regions in revised text in Line 134-136 (revised version with track changes). We analyzed the numbers of upregulated and downregulated TEs covered by H2Aub or H3K27me3 in h2a_ub and Mpbmi1/1l mutants (Fig. 2k and 3o). Around 20% of deregulated TEs are covered by H2Aub in h2a_ub and Mpbmi1/1l mutants. As the reviewer suggested, we found H2Aub to be decreased on the downregulated TEs in h2a_ub mutants, supporting that H2Aub is required for TE activation.*

*(iv) We added information about H2Aub levels on TEs commonly downregulated in h2a_ub and Mpbmi1/1l mutants as metaplots, boxplots and IGV profiles (Fig. 5h, 5i, S11).*

In brief, while the authors illustrated an interplay between H2Aub and PRC2 mediated H3K27me3 over genic and intergenic regions, this would be better substantiated by further graphic representation of the results. As mentioned above, i figure 6a to 6d the authors should again use a similar scale to better appreciate the differences between the different classes, as well as stating clearly the number of genes/regions underlying the different plots. As for figure 1l the authors could further substantiate these conclusions by plotting the level of H3K27me3 over all peaks, or all genes, or all intergenic regions to underling if the influence of PRC1 mutation is limited over the H2Aub/H3K27me3 overlapping regions or rather a general genome wide influence. Heatmap profile of the different regions would also help appreciating if the changes of marking are specifically located over gene promoters or rather span over the all annotation of genes/ intergenic regions. The IGV profiles are quite zoomed in to being able to appreciate the quality of the Chip-seq data, further regions and zoom out profile would help illustrating how the peaks are separated from the background noise. Also adding a track with genes, or IGV screenshot of representative genes, would help appreciating the differences in marking between genic regions and intergenic regions and further help to corroborate the conclusions.

*Reply: (i) We applied the same scales in Fig. 6a to 6d and found that H2Aub/H3K27me3 marked genes have higher H2Aub levels compared to only-H2Aub genes. We included the number of genes for each category on top of the plots.*

*(ii) We tested H3K27me3 levels on all genes and all TEs (instead of intergenic regions) in Fig. S5 and found that H3K27me3 levels were generally decreased on all genes but not on all TEs, suggesting that H2Aub has a general effect on the H3K27me3 incorporation in genic region, but not in TE regions, where reduced H3K27me3 was only observed on the H2Aub/H3K27me3 double marked TEs.*

*(iii) We included heatmaps of H3K27me3 on all genes and TEs in WT and h2a_ub mutants in Fig. S5.*

*(iv) We included IGV profiles in Fig. S3c and S3d.*

Indeed, in the discussion the authors drive some strong conclusion, such as that PRC1-catalysed H2Aub is required for TE repression in Marchantia (line 237) without exploring the role over TE expression and without showing a specific link of changes of H2Aub or H3K27me3 level over the TE upregulated. These examples illustrate the need of better substantiate and more clearly link the conclusions with the presentation of the results.

*Reply: Thanks for pointing this out. We specifically analyzed H2Aub and H3K27me3 on up- and downregulated TEs and found that PRC1-mediated H2Aub is required for TE activation, but not for repression. We included this information in Fig. 2m-2p, 5h and 5i and made corresponding changes in the discussion in Line 279-282 (revised version with track changes).*

Minor points:

- How look like the Marchantia H2A protein, as compared to the different H2A variants found in Arabidopsis (H2A.Z, H2A.W, H2A.X). In particular, it would be interesting to test H2A.Z ubiquitination in the Mpbmi1/1l mutant line to gain a better understanding of its phenotype.

a. Multiple sequence alignments by ClustalX. The ubiquitinated lysine residues in MpH2A are pointed by purple arrowhead. b. Arabidopsis H2A.Z and H2A.Zub probed by anti-HTA9 antibody in western blot.

*Reply: The Marchantia H2A protein (MpH2A) shares high sequence similarity with Arabidopsis proteins H2A.Z, H2A.W and H2A.X (AtH2A.Z, AtH2A.W and AtH2A.X, see Fig. a above). Due to lack of a suitable antibody against H2A.Z (or H2A.W, H2A.X) ubiquitination, we could not test H2A.Zub in the Mpbmi1/1l mutants. The anti-HTA9 antibody against AtH2A.Z recognizes AtH2A.Zub in Arabidopsis (see Fig. b above), but not in Marchantia.*

- When citing literature, please better highlight when describing situations in Marchantia or in other species such as Arabidopsis. This can be confusing at different places along the manuscript.

*Reply: Thanks for pointing this out. We made corresponding changes in Line 69, 72, 93, 269, 290, 295, 302 and 305 (revised version with track changes).*

- In Figure1 there are large differences of scales in the boxplots of gene categories "all marked genes" and "H2Aub/H3K27me3 marked genes" as compared with the "intergenic regions". Using the same scale for all boxplots would better illustrate the amplitude of the different changes.

*Reply: We applied the same scales for gene and TE regions in Fig. 1m, 1o, 6a-6d, 6g and 6h.*

- Minor mistake, figure 5e seems to represent a Venn comparison of overlap between 5 and 6 (and not 3 and 4)

*Reply: Thanks for pointing this out. We corrected this in Fig. 5e.*

- Police size in the figures is well too small

*Reply: We adjusted the fond size in the figures.*

**Second round of review**

**Reviewer 1**

The authors have addressed most of my concerns with the previous version of the manuscript. After reading the revised manuscript, I have a few new questions that authors may consider to address.

1. In fig6e, it is stated that 1,388 genes showed reduced H2Aub in the Mpbmi1/1l mutant, I would suggest the authors to indicate such H2Aub reduced gene numbers in fig1i and 1j.

2. In fig1i, 1j, 6e, S3a and S3b, genes losing H2Aub seem to carry low levels of H3K27me3 when compared with H3K27me3-marked genes (fig1m, 6c, 6d, S3e, S12c and S12d). How many of these genes that lost H2Aub are H3K27me3-marked genes? Do genes losing H2Aub carry H3K27me3 above the ChIP-seq background level? I am asking this question because it might not be meaningful to compare H3K27me3 levels at these loci if most of them do not carry real H3K27me3 signal.

3. It's interesting that in H2A point mutated mutants, the only H3K27me3-marked genes (636 genes) showed reduced H3K27me3 but strongly increased H2Aub (figS3e and 3f). A possible explanation proposing H3K27 demethylation activity at these loci was provided. The authors are suggested to show the H2Aub levels at genes enriched with both H2Aub and H3K27me3 (1,260 genes) (box plot and metagene plot), which if I understand the conclusion correctly (H2Aub is required for H3K27me3), is suppose to be reduced in H2A point mutated mutants.

4. In fig6d and figS12d, the only H3K27me3-marked genes (636 genes) showed no defects of H3K27me3 in the Mpbmi1/1l mutant. The authors are suggested to show the H2Aub levels at these loci, and compare with that in H2A point mutated mutants.

5. Line 267, the authors claim that loss of Mpbmi1/1l impaired genome-wide deposition of H3K27me3. But based on results showed in fig6c and 6d, at least 1/3 of the H3K27me3-marked genes (only-H3K27me3 genes) are not affected. The authors may consider revising their statement or providing more evidence to support the genome-wide reduction of H3K27me3 in the Mpbmi1/1l mutant.

**Reviewer 2**

This is an interesting study for the readership of Genome Biology.

**Authors Response**

**Point-by-point responses to the reviewers’ comments:**

We would like to thank the reviewer and editor for the constructive comments.

We found that our lists of genes marked by only H2Aub and only H3K27me3 contained duplicated gene IDs, caused by different H2Aub or H3K27me3 peaks annotated to the same gene. We made corresponding changes in Fig. 1l, 2c, 2d, 3m, 6a, 6d, 6e, S3g, S12a and S12d. The minor changes in numbers do not influence the results and conclusions.

We also noticed a mistake in the calculations underlying Fig. S3h (previous Fig. S3f). The result showed that H2Aub level was highly increased on the gene body but mildly increased on the promoter of only-H3K27me3 genes in h2a_ub mutants. This correction does not affect our conclusions.

**Reviewer #1:**

The authors have addressed most of my concerns with the previous version of the manuscript. After reading the revised manuscript, I have a few new questions that authors may consider to address.

1. In fig6e, it is stated that 1,388 genes showed reduced H2Aub in the Mpbmi1/1l mutant, I would suggest the authors to indicate such H2Aub reduced gene numbers in fig1i and 1j.

*Reply: We added the numbers of genes losing H2Aub in h2a_ub mutants in Fig. 1i and 1j.*

2. In fig1i, 1j, 6e, S3a and S3b, genes losing H2Aub seem to carry low levels of H3K27me3 when compared with H3K27me3-marked genes (fig1m, 6c, 6d, S3e, S12c and S12d). How many of these genes that lost H2Aub are H3K27me3-marked genes? Do genes losing H2Aub carry H3K27me3 above the ChIP-seq background level? I am asking this question because it might not be meaningful to compare H3K27me3 levels at these loci if most of them do not carry real H3K27me3 signal.

*Reply: Genes losing H2Aub in Mpbmi1/1l and h2a_ub mutants significantly overlap with genes marked by H3K27me3 above background level (Fig. S12g-S12i). We therefore consider it meaningful to show this data.*

3. It's interesting that in H2A point mutated mutants, the only H3K27me3-marked genes (636 genes) showed reduced H3K27me3 but strongly increased H2Aub (figS3e and 3f). A possible explanation proposing H3K27 demethylation activity at these loci was provided. The authors are suggested to show the H2Aub levels at genes enriched with both H2Aub and H3K27me3 (1,260 genes) (box plot and metagene plot), which if I understand the conclusion correctly (H2Aub is required for H3K27me3), is supposed to be reduced in H2A point mutated mutants.

*Reply: We added box- and metagene plots showing H2Aub levels on H2Aub/H3K27me3 genes in Fig. S3f. Due to the compensation of ubiquitination of K115/K116 and K119 in H2A, a mild, but significant decrease of H2Aub was only observed on the promoter of H2Aub/H3K27me3 genes (Fig. S3f) in h2a_ub mutants, supporting the idea that H2Aub is essential for H3K27me3 deposition on the promoter of H2Aub/H3K27me3 genes. In the Mpbmi1/1l mutant, the H2Aub and H3K27me3 levels were significantly decreased on H2Aub/H3K27me3 genes in both, the promoter and gene body, supporting our conclusion that H2Aub is essential for H3K27me3 deposition. H3K27me3 was also decreased on only H3K27me3 genes in h2a_ub mutants; however, on H3K27me3 only genes the H2Aub level was not decreased, but mildly increased on the promoter, and highly increased on the gene body (Fig. S3h, a corrected version of previous Fig. S3f). In the Mpbmi1/1l mutant, H2Aub and H3K27me3 did not show obvious changes on only H3K27me3 genes (Fig. S12d-S12f).*

4. In fig6d and figS12d, the only H3K27me3-marked genes (636 genes) showed no defects of H3K27me3 in the Mpbmi1/1l mutant. The authors are suggested to show the H2Aub levels at these loci, and compare with that in H2A point mutated mutants.

*Reply: We examined H2Aub level on only H3K27me3 genes in Mpbmi1/1l mutants and found it does not change in the Mpbmi1/1l mutants compared to WT (Fig. S12e, S12f), consistent with the unchanged H3K27me3 level. In H2Aub point mutated lines, gain of H2Aub is correlated with decreased H3K27me3 level on only H3K27me3 genes (see previous comment).*

5. Line 267, the authors claim that loss of Mpbmi1/1l impaired genome-wide deposition of H3K27me3. But based on results showed in fig6c and 6d, at least 1/3 of the H3K27me3-marked genes (only-H3K27me3 genes) are not affected. The authors may consider revising their statement or providing more evidence to support the genome-wide reduction of H3K27me3 in the Mpbmi1/1l mutant.

*Reply: We agree and revised our statement to “Loss of MpBMI1/1L activity in Marchantia impaired H3K27me3 deposition on H2Aub and H3K27me3 marked genes” in the second paragraph of discussion.*

*To address editorial concerns, we added a reference for GEO datasets which is publicly available now, provided full uncropped western blots in Fig. S13, renamed the supplemental file to "additional file” and cited them explicitly, added Ethical Approval section, added a subsection listing all the additional files and included the edited abstract. Since one edited sentence in the abstract changed our meaning, “These mutants show reduced H3K27me3 levels on the same target sites as they are defective in PRC1 subunits MpBMI1 and the homolog MpBMI1L”, we kept our previous version.*
